# Supplementary material for: Quantum advantage and stability to errors in analogue quantum simulators
Source: Nat Commun. 2024 Aug 2;15:6507. doi: 10.1038/s41467-024-50750-x (PMC11297267; doi:10.1038/s41467-024-50750-x)
Supplement: Supplementary file 1 — Supplementary Information [file 41467_2024_50750_MOESM1_ESM.pdf]

# Supplement to "Quantum advantage and stability to errors in analogue quantum simulators"

Rahul Trivedi,[1,2,3,*] Adrian Franco Rubio,[1,2,*] and J. Ignacio Cirac[1,2]

[1] *Max-Planck-Institut für Quantenoptik, Hans-Kopfermann-Str. 1, 85748 Garching, Germany.*
[2] *Munich Center for Quantum Science and Technology (MCQST), Schellingstr. 4, D-80799 Munich, Germany.*
[3] *Electrical and Computer Engineering, University of Washington, Seattle, WA - 98195, USA*

(Dated: July 3, 2024)

## Supplemental note I: Notation and preliminaries

The following is a list of notations that we will use for the mathematical proofs in the following appendices. Here $v$ denotes a vector, and $A$ a matrix.

- $\|v\|_p \equiv (\sum_i v_i^p)^{\frac{1}{p}}$ denotes the $p$-norm of the vector $v$. In the $p \to \infty$ limit, it becomes the max norm, $\|v\|_\infty \equiv \max_i |v_i|$.
- $\|A\|_{\text{op},p}$ is the $p^{\text{th}}$ Schatten norm of $A$ i.e. the $p-$norm of the singular values of $A$. Note that $\|A\|_{\text{op}} := \|A\|_{\text{op},\infty}$ is also the operator norm of $A$, i.e. the $\|A\|_{\text{op},\infty} = \sup_{x, \|x\|_2 = 1} \|Ax\|_2$ and $\|A\|_{\text{op},1}$ denotes the trace norm, i.e. the 1-Schatten norm of $A$.
- For a superoperator $\mathcal{A}$, we define the $\|\mathcal{A}\|_{p\to p} = \max_{O, \|O\|_{\text{op},p}=1} \|\mathcal{A}(O)\|_{\text{op},p}$. We define the completely bounded version of this norm, $\|\mathcal{A}\|_{p\to p, cb} = \sup_{n\geq 2} \|\mathcal{A} \otimes \text{id}_n\|_{p\to p}$, which is stable under tensor product. Furthermore, as is standard, we will use $\|\mathcal{A}\|_\diamond := \|\mathcal{A}\|_{1\to 1, cb}$.
- vec$(A)$ denotes the vectorization of $A$, i.e. the vector whose components are the matrix elements of $A$. The precise order in which the matrix elements are arranged in the vector will not be relevant for our proofs, and can be arbitrarily chosen.
- Unless otherwise mentioned, $\|v\|$, where $v$ is a vector, will denote its $\ell^2$ norm and $\|O\|$, where $O$ is an operator, will be its operator norm.

In our analysis of Gaussian fermion models, we will assume that for each site $x \in \mathbb{Z}_L^d$, there are $D$ fermionic modes. Suppose $a_x^n$, for $x \in \mathbb{Z}_L^d$ and $n \in \{1, 2 \dots D\}$ be the fermionic annihilation operator corresponding to the $n^{\text{th}}$ fermion mode at the site $x$. The Majorana operators, $c_x^{2n-1}, c_x^{2n}$ associated with this mode will be

$$c_x^{2n-1} = \frac{1}{\sqrt{2}}\big(a_x^{n\dagger} + a_x^n\big) \text{ and } c_x^{2n} = \frac{1}{\sqrt{2}i}\big(a_x^{n\dagger} - a_x^n\big).$$

In most of the proofs, we will not need to distinguish between the two Majorana operators and will represent them as $c_x^\alpha$ with the index $\alpha \in \{1, 2 \dots 2D\}$. For a Hermitian operator $O$ expressed as a quadratic form over the Majorana operators $c_x^\alpha$,

$$O = \sum_{x,y \in \mathbb{Z}_L^d} \sum_{\alpha,\beta=1}^{2D} o_{x,y}^{\alpha,\beta} c_x^\alpha c_y^\beta, \tag{1}$$

we will denote by $\tilde{O}$ the matrix of coefficients $o_{x,y}^{\alpha,\beta}$, with the indices $(x, \alpha)$ corresponding to the rows and $(y, \beta)$ corresponding to the columns. We will assume, without loss of generality and unless otherwise mentioned, that $\tilde{O}$ is a Hermitian matrix with purely imaginary matrix elements.

Unless otherwise mentioned, $n$ will be used for the number of spins or fermionic modes in the lattice system under consideration. For Gaussian fermionic models considered in this paper, defined on the lattice $\mathbb{Z}_L^d$ and with $D$ fermions per site, we will use $N = L^d$ to denote the number of lattice sites and therefore $n = DN$.

We will need the following two lemmas:

---

* Both authors contributed equally.

*Lemma* 1. Let $M \in \mathbb{C}^{n\times m}$ such that $|M_{ij}| \leq \delta, \forall i, j$, the rows of $M$ have at most $m_r$ nonzero elements and the columns of $M$ have at most $m_c$ nonzero elements, then $\|M\|_{\text{op}} \leq \sqrt{m_c m_r}\delta$.

*Proof.* Let $v \in \mathbb{C}^m$ be a vector, and denote $\mathcal{R}_i \equiv \{j | M_{ij} \neq 0\}$, $\mathcal{C}_j \equiv \{i | M_{ij} \neq 0\}$. Note that, by assumption, $|\mathcal{R}_i| \leq m_r \ \forall i, |\mathcal{C}_j| \leq m_c \ \forall j$. Then,

$$\begin{aligned}
\|Mv\|^2 &= \sum_{i=1}^n \left| \sum_{j\in\mathcal{R}_i} M_{ij} v_j \right|^2 \\
&\leq \sum_{i=1}^n \left( \sum_{j\in\mathcal{R}_i} |M_{ij}|^2 \sum_{k\in\mathcal{R}_i} |v_k|^2 \right) \\
&\leq \sum_{i=1}^n |\mathcal{R}_i| \delta^2 \sum_{k\in\mathcal{R}_i} |v_k|^2 \leq m_r \delta^2 \sum_{i=1}^n \sum_{k\in\mathcal{R}_i} |v_k|^2 \\
&= m_r \delta^2 \sum_{k=1}^n \sum_{i\in\mathcal{C}_k} |v_k|^2 = m_r \delta^2 \sum_{k=1}^n |\mathcal{C}_k| |v_k|^2 \\
&\leq m_r m_c \delta^2 \|v\|^2 \implies \|M\|_{\text{op}} \leq \sqrt{m_r m_c}\delta.
\end{aligned}$$

*Lemma* 2. Given bounded Hermitian operators $H$ and $H'$, for any bounded operator $O$

$$\left\| e^{iH't} O e^{-iH't} - e^{iHt} O e^{-iHt} \right\|_{\text{op}} \leq 2\|O\|_{\text{op}} \|H - H'\|_{\text{op}} t.$$

*Proof.* Consider the operator $\tilde{O}(t) \equiv e^{-iHt} e^{iH't} O e^{-iH't} e^{iHt}$. Note that

$$\frac{d}{dt}\tilde{O}(t) = i\Big[ e^{-iHt}(H' - H) e^{iH't} O e^{-iH't} e^{iHt} - e^{-iHt} e^{iH't} O e^{-iH't} (H' - H) e^{iHt} \Big],$$

and consequently,

$$\left\| \frac{d}{dt}\tilde{O}(t) \right\| \leq 2\|H - H'\| \|O\|.$$

We then immediately obtain that

$$\left\| e^{iH't} O e^{-iH't} - e^{iHt} O e^{-iHt} \right\| \leq \int_0^t \left\| \frac{d}{ds}\tilde{O}(s) \right\| ds \leq 2\|O\| \|H - H'\| t.$$

## Supplemental note II: Gaussian fermion models

### A. Proof of proposition 1 (Dynamics of Gaussian fermion models)

We first derive an equation of motion for the correlation matrix $\Gamma$ of the Gaussian fermionic quantum simulator model, including the non-Markovian decohering errors, described in the main text. It is convenient to consider a system-environment Hamiltonian $\hat{H}(t)$

$$\hat{H}(t) = H + \sum_{j,x} \Big( B_{j,x}^\dagger(t) L_{j,x} + L_{j,x}^\dagger B_{j,x}(t) \Big) + \sum_{j,x} \Big( A_{j,x}^\dagger(t) Q_{j,x} + Q_{j,x}^\dagger A_{j,x}(t) \Big),$$

where $\{B_{j,x}(t), B_{j',x'}^\dagger(t')\} = \delta_{x,x'}\delta_{j,j'}\delta(t - t')$ and $\{A_{j,x}(t), A_{j',x'}^\dagger(t')\} = \delta_{j,j'}\delta_{x,x'} K_{j,x}(t - t')$. Furthermore, we also assume that the environments corresponding to the operators $B_{j,x}(t)$ and $A_{j,x}(t)$ are independent by enforcing $\{A_{j,x}(t), B_{j',x'}^\dagger(t')\} = 0$. Since the operators $B_{j,x}(t)$ are delta function correlated, it can easily be verified that

tracing out the environment correspnding to these operators effectively yields a master equation with jump operators $L_{j,x}$. Now, we derive a set of dynamical equations for the correlation matrix

$$(\Gamma(t))^{\alpha,\beta}_{x,y} = \frac{1}{2}\text{Tr}\big([c^\alpha_x, c^\beta_y]\rho(t)\big) = \frac{1}{2}\text{Tr}\big([c^\alpha_x(t), c^\beta_y(t)]\rho(0)\big) = \text{Tr}\big(c^\alpha_x(t)c^\beta_y(t)\rho(0)\big) - \frac{1}{2}\delta_{\alpha,\beta}\delta_{x,y}.$$

where $c^\alpha_x(t) = \hat{U}(0,t)c^\alpha_x\hat{U}(t,0)$, with $\hat{U}(t,s)$ being the propagator corresponding to $\hat{H}(t)$ and $\rho(0)$ is the initial system-environment state, which we assume to be vacuum for the environment. It is convenient to introduce the two-point correlation matrix

$$\big(\Lambda(t,s)\big)^{\alpha,\beta}_{x,y} = \text{Tr}\big(c^\alpha_x(t)c^\beta_y(s)\rho(0)\big).$$

It can be noted that $\Gamma(t) = \Lambda(t,t) - I_{2n}/2$ and $\Lambda^T(t,t) = I_{2n} - \Lambda(t,t)$.

*Lemma* 3. Assuming that the environment is initially in the vacuum state, the correlation matrix $\Gamma(t)$ satisfies,

$$\frac{d}{dt}\Gamma(t) = X\Gamma(t) + \Gamma(t)X^{\text{T}} + Y + Z(t),$$

where

$$\begin{aligned}
X &= -i\tilde{H} - \frac{1}{2}(\tilde{Q} + \tilde{Q}^*),\\
Y &= \frac{1}{2}(\tilde{Q} - \tilde{Q}^*),\\
Z(t) &= \frac{1}{2}\int_0^t \Big(\tilde{L}^*(s-t)\Lambda^{\text{T}}(t,s) + \Lambda^{\text{T}}(t,s)\tilde{L}^\dagger(s-t) - \tilde{L}(t-s)\Lambda(s,t) - \Lambda(s,t)\tilde{L}^{\text{T}}(t-s)\Big)ds,
\end{aligned}$$

with $\tilde{Q}, \tilde{L}(\tau) \in \mathbb{C}^{2n\times 2n}$ being given by,

$$\big(\tilde{Q}\big)^{\alpha,\beta}_{x,y} = \sum_{j,z} q^\alpha_{j,z;x}q^{\beta^*}_{j,z;y} \text{ and } \big(\tilde{L}(\tau)\big)^{\alpha,\beta}_{x,y} = \sum_{j,z} K_{j,z}(\tau)l^\alpha_{j,z;x}l^{\beta^*}_{j,z;y}.$$

*Proof*: Our starting point are the Heisenberg equations of motion for $c^\alpha_x(t)$,

$$i\frac{d}{dt}c^\alpha_x(t) = 2\sum_{y,\beta} h^{\alpha,\beta}_{x,y}c^\beta_y(t) + \sum_{j,z}\Big(q^{\alpha*}_{j,z;x}A_{j,z}(t;t) - q^\alpha_{j,z;x}A^\dagger_{j,z}(t;t)\Big) + \sum_{j,z}\Big(l^{\alpha*}_{j,z;x}B_{j,z}(t;t) - l^\alpha_{j,z;x}B^\dagger_{j,z}(t;t)\Big), \quad (2)$$

where $A_{j,z}(\tau;t) = U(0,t)A_{j,z}(\tau)U(t,0)$ and $B_{j,z}(\tau;t) = U(0,t)B_{j,z}(\tau)U(t,0)$. Furthermore, we can also obtain and integrate the Heisenberg equations of motion for $A_{j,z}(\tau;t)$ and $B_{j,z}(\tau;t)$ to obtain

$$\begin{aligned}
&\frac{d}{dt}A_{j,z}(\tau;t) = -i\sum_{\beta,y} q^\beta_{j,z;y}c^\beta_y(t)\delta(t-\tau) \implies A_{j,z}(t;t) = A_{j,z}(t) - \frac{i}{2}\sum_{\beta,y} q^\beta_{j,z;y}c^\beta_y(t) \text{ and}\\
&\frac{d}{dt}B_{j,z}(\tau;t) = -i\sum_{\beta,y} l^\beta_{j,z;y}c^\beta_y(t)K_{j,z}(\tau-t) \implies B_{j,z}(t;t) = B_{j,z}(t) - i\sum_{\beta,y} q^\beta_{j,z;y}\int_0^t K_{j,z}(t-s)c^\beta_y(s)ds.
\end{aligned}$$

Since $\rho(0)$ is assumed to be in the vacuum state in the environments corresponding to annihilation operators $A_{j,x}(t)$ and $B_{j,x}(t)$, it then follows that

$$A_{j,z}(t;t)\rho(0) = -\frac{i}{2}\sum_{\beta,y} q^\beta_{j,z;y}c^\beta_y(t)\rho(0) \text{ and } B_{j,z}(t;t)\rho(0) = -i\sum_{\beta,y} q^\beta_{j,x;y}\int_0^t K_{j,z}(t-s)c^\beta_y(s)\rho(0)ds. \quad (3)$$

Now, we can obtain a differential equation for the correlation matrix element $(\Gamma(t))^{\alpha,\beta}_{x,y}$ — from its definition, it follows that

$$\begin{aligned}
\frac{d}{dt}\big(\Gamma(t)\big)^{\alpha,\beta}_{x,y} &= \frac{1}{2}\text{Tr}\Big(\frac{d}{dt}c^\alpha_x(t)c^\beta_y(t)\rho(0)\Big) + \frac{1}{2}\text{Tr}\Big(c^\alpha_x(t)\frac{d}{dt}c^\beta_y(t)\rho(0)\Big),\\
&= -i\big([\tilde{H}, \Gamma(t)]\big)^{\alpha,\beta}_{x,y} + \frac{i}{2}\sum_{j,z}\Big(q^\alpha_{j,z;x}\text{Tr}\big(c^\beta_y(t)\rho(0)A^\dagger_{j,z}(t;t)\big) + q^{\alpha^*}_{j,z;x}\text{Tr}\big(c^\beta_y(t)A_{j,z}(t;t)\rho(0)\big)\Big).
\end{aligned}$$

Furthermore, using Eqs. 2 and 3, we obtain that

$$
\begin{aligned}
&\mathrm{Tr}\bigg(\frac{d}{dt}c_x^\alpha(t)c_y^\beta(t)\rho(0)\bigg)\\
&\quad= -i\big(\tilde{H}\Lambda(t,t)\big)_{x,y}^{\alpha,\beta} + i\sum_{j,z}\bigg(q_{j,z;x}^\alpha \mathrm{Tr}\big(c_y^\beta(t)\rho(0)A_{j,z}^\dagger(t;t)\big) + q_{j,z;x}^{\alpha^*}\mathrm{Tr}\big(c_y^\beta(t)A_{j,z}(t;t)\rho(0)\big)\bigg)\\
&\qquad + i\sum_{j,z}\bigg(l_{j,z;x}^\alpha \mathrm{Tr}\big(c_y^\beta(t)\rho(0)B_{j,z}^\dagger(t;t)\big) + l_{j,z;x}^{\alpha^*}\mathrm{Tr}\big(c_y^\beta(t)B_{j,z}(t;t)\rho(0)\big)\bigg),\\
&\quad= -i\big(\tilde{H}\Lambda(t,t)\big)_{x,y}^{\alpha,\beta} - \frac{1}{2}\sum_{j,z,\alpha',x'}\bigg(q_{j,z;x}^\alpha q_{j,z;x'}^{\alpha'^*}\mathrm{Tr}\big(c_{x'}^{\alpha'}(t)c_y^\beta(t)\rho(0)\big) - q_{j,z;x}^{\alpha^*}q_{j,z;x'}^{\alpha'}\mathrm{Tr}\big(c_y^\beta(t)c_{x'}^{\alpha'}(t)\rho(0)\big)\bigg)\\
&\qquad - \frac{1}{2}\sum_{j,z,\alpha',x'}\int_0^t K_{j,z}(t-s)\bigg(l_{j,z;x}^\alpha l_{j,z;x'}^{\alpha'^*}\mathrm{Tr}\big(c_{x'}^{\alpha'}(s)c_y^\beta(t)\rho(0)\big) - l_{j,z;x}^{\alpha^*}l_{j,z;x'}^{\alpha'}\mathrm{Tr}\big(c_y^\beta(t)c_{x'}^{\alpha'}(s)\big)\bigg)ds,\\
&\quad= \bigg(-i\tilde{H}\Lambda(t,t) - \frac{1}{2}\big(\tilde{Q}\Lambda(t,t) - \tilde{Q}^*\Lambda^{\mathrm{T}}(t,t)\big) - \frac{1}{2}\int_0^t\big(\tilde{L}(t-s)\Lambda(s,t) - \tilde{L}^*(s-t)\Lambda^{\mathrm{T}}(t,s)\big)ds\bigg)_{x,y}^{\alpha,\beta}, \qquad (4)
\end{aligned}
$$

A similar manipulation yields that

$$
\begin{aligned}
&\mathrm{Tr}\bigg(c_x^\alpha(t)\frac{d}{dt}c_y^\beta(t)\rho(0)\bigg)\\
&\quad= \bigg(-i\Lambda(t,t)\tilde{H}^{\mathrm{T}} - \frac{1}{2}\big(\Lambda(t,t)\tilde{Q}^{\mathrm{T}} - \Lambda^{\mathrm{T}}(t,t)\tilde{Q}^\dagger\big) - \frac{1}{2}\int_0^t\big(\Lambda(s,t)\tilde{L}^{\mathrm{T}}(t-s) - \Lambda^{\mathrm{T}}(t,s)\tilde{L}^\dagger(s-t)\big)ds\bigg)_{x,y}^{\alpha,\beta}. \qquad (5)
\end{aligned}
$$

From Eqs. 4 and 5, together with the facts that $\Lambda(t,t) = \Gamma(t) + I_{2n}/2, \Lambda^{\mathrm{T}}(t,t) = -\Gamma(t) + I_{2n}/2$, we then obtain the dynamical equation for $\Gamma(t)$. □

Suppose that in the absence of any errors and noise, our target is to implement a spatially local Hamiltonian $H$ and jump operators $Q_{j,x}$. In the presence of errors and noise, we instead implement a perturbed Hamiltonian $H'$ described by coefficients $h_{x,y}^{\alpha,\beta'}$, perturbed jump operators $Q'_{j,x}$ described by coefficients ${q'}_{j,x;y}^{\alpha}$, as well as interaction with a decohering environment captured by the operators $L_{j,x}$ described by coefficients $l_{j,x;y}^\alpha$ which satisfy

$$
|h_{x,y}^{\alpha,\beta} - {h'}_{x,y}^{\alpha,\beta}| \leq \delta, |q_{j,x;y}^\alpha - {q'}_{j,x;y}^{\alpha}| \leq \delta \text{ and } |l_{j,x;y}^\alpha| \leq \sqrt{\delta}.
$$

*Lemma* 4. Let $a_{x,\alpha}^{y,\beta}, {a'}_{x,\alpha}^{y,\beta} \in \mathbb{C}$ for $x, y \in \mathbb{Z}_L^d$, $\alpha, \beta \in \{1, 2 \ldots 2D\}$ satisfy $a_{x,\alpha}^{y,\beta}, {a'}_{x,\alpha}^{y,\beta} = 0$ if $d(x,y) > R$ and $b_{j,x;y}^\alpha, {b'}_{j,x;y}^{\alpha} \in \mathbb{C}$ for $x, y \in \mathbb{Z}_L^d$, $j \in \{1, 2 \ldots n_L\}, \alpha \in \{1, 2 \ldots 2D\}$ be such that $b_{j,x;y}^\alpha, {b'}_{j,x;y}^{\alpha} = 0$ if $d(x,y) > R$. Furthermore, $\exists a_0 > 0 : |a_{x,\alpha}^{y,\beta}| \leq a_0\ \forall x, y, \alpha, \beta$, $\exists b_0 > 0 : |b_{j,x;y}^\alpha| \leq b_0\ \forall x, y, j, \alpha$ and $\exists \delta > 0 : |a_{x,\alpha}^{y,\beta} - {a'}_{x,\alpha}^{y,\beta}|, |b_{j,x;y}^\beta - {b'}_{j,x;y}^{\beta}| \leq \delta\ \forall x, y, j, \alpha, \beta$. Denote by $A, A' \in \mathbb{C}^{2n\times 2n}$ the matrices formed by $a_{x,\alpha}^{y,\beta}, {a'}_{x,\alpha}^{y,\beta}$ with $(x,\alpha)$ corresponding to the rows and $(y,\beta)$ corresponding to the columns, and by $B, B' \in \mathbb{C}^{n_L N\times 2n}$ the matrices formed by $b_{j,x;y}^\alpha, {b'}_{j,x;y}^{\alpha}$ with $(j,x)$ corresponding to the rows and $(y,\alpha)$ corresponding to the columns. Then,

$$
\begin{aligned}
&\|A\| \leq 2D(2R+1)^d a_0, \|B\| \leq \sqrt{2Dn_L}(2R+1)^d b_0,\\
&\|A - A'\| \leq 2D(2R+1)^d\delta, \|B^\dagger B - B'^\dagger B'\| \leq 4Dn_L(2R+1)^{2d}(2b_0\delta + \delta^2).
\end{aligned}
$$

*Proof.* The proof of this lemma is a repeated application of lemma 1. We note that the matrices $A, A'$ have at most $2D(2R+1)^d$ non-zero element in any row or column. The matrices $B, B'$ have at most $n_L(2R+1)^d$ non-zero elements in their columns and at most $2D(2R+1)^d$ non-zero elements in their rows. Thus, from lemma 1, we obtain the bounds

$$
\|A\| \leq 2D(2R+1)^d a_0, \|A'\| \leq 2D(2R+1)^d a_0', \|B\| \leq \sqrt{2Dn_L}(2R+1)^d b_0, \|B'\| \leq \sqrt{2Dn_L}(2R+1)^d b_0'.
$$

where $a_0' = a_0 + \delta$ and $b_0' = b_0 + \delta$ are upper bounds on the coefficients $|{a'}_{x,\alpha}^{y,\beta}|$ and $|{b'}_{j,x;y}^{\alpha}|$ respectively. Since $A - A'$ also has at most $2D(2R+1)^d$ non-zero element in any row or column, it immediately follows that

$$
\|A - A'\| \leq 2D(2R+1)^d\delta.
$$

Furthermore, since $B - B'$ has at most $n_L(2R+1)^d$ non-zero elements in its columns and at most $2D(2R+1)^d$ non-zero elements in its rows

$$\|B - B'\| \leq \sqrt{2Dn_L}(2R+1)^d\delta.$$

We can now estimate $\|B^\dagger B - B'^\dagger B'\|$:

$$\begin{aligned}\|B^\dagger B - B'^\dagger B'\| &= \|B^\dagger(B - B') + (B^\dagger - B'^\dagger)B'\|,\\ &\leq (\|B\| + \|B'\|)\|B - B'\|,\\ &\leq 4Dn_L(2R+1)^{2d}(2b_0\delta + \delta^2).\end{aligned}$$

Finally, we provide an upper bound on $Z(t)$ defined in lemma 3 — for this, we assume an upper bound on the kernels are $K_{j,z}(\tau) = \{B_{j,z}(\tau), B^\dagger_{j,z}(0)\}$. We consider kernels which can also contain delta functions i.e. $K_{j,z}(\tau)$ to be of the form

$$K_{j,z}(\tau) = K^c_{j,z}(\tau) + \sum_{i=1}^{M} k^i_{j,z}\delta(\tau - \tau_i),$$

where $K^c_{j,z}(\tau)$ is a continous function of $\tau$. Then, we define a kernel $K(\tau)$ by

$$K(\tau) = K^c(\tau) + \sum_{i=1}^{M} k^i\delta(\tau - \tau_i) \text{ where } K^c(\tau) = \sup_{j,z}|k^c_{j,z}(\tau)| \text{ and } k^i = \sup_{j,z}|k^i_{j,z}|.$$

The kernel $K(\tau)$ can be considered as a distributional upper bound on $K_{j,z}(\tau)$ i.e. for any continuous and compactly supported function $f$,

$$\left|\int_{\mathbb{R}} K^c_{j,z}(\tau)f(\tau)d\tau\right| \leq \int_{\mathbb{R}} K(\tau)|f(\tau)|d\tau.$$

In the following lemma, under the assumption that $K(\tau)$ has a bounded integral, we provide an upper bound on $Z(t)$.

*Lemma* 5. If $\int_{\mathbb{R}} K(\tau)d\tau \leq 1$ and $|l^\alpha_{j,z;x}| \leq \sqrt{\delta}$, then $\|Z(t)\| \leq 2\sqrt{2Dn_L}(2R+1)^d\delta$, where $Z(t)$ is defined in lemma 3.

*Proof.* First, we will establish that $\|\Lambda(t,s)\| \leq 1$ for any $t, s$. For this, we consider two vectors $v, u \in \mathbb{C}^{2n}$ and upper bound $|v^\dagger\Lambda(t,s)u|$. From the definition of $\Lambda(t,s)$, we have that

$$|v^\dagger\Lambda(t,s)u| = \left|\sum_{x,y}\sum_{\alpha,\beta=1}^{2D} v^{\alpha^*}_x \mathrm{Tr}[c^\alpha_x(t)c^\beta_y(s)\rho(0)]u^\beta_y\right| \leq \left|\mathrm{Tr}(c^\dagger_v(t)c_u(s)\rho(0))\right| \leq \|c_v(t)\|\|c_u(s)\| = \|c_v\|\|c_u\|, \tag{6}$$

where we have defined the operator $c_v = \sum_x\sum_{\alpha=1}^{2D} v^\alpha_x c^\alpha_x$. Now, to bound $\|c_v\|$, we note that $\{c_v, c^\dagger_v\} = \|v\|^2 I$, and therefore

$$c^\dagger_v c_v = \|v\|^2 I - c_v c^\dagger_v \prec \|v\|^2 I \implies \|c_v\| \leq \|v\|. \tag{7}$$

Therefore, from Eqs. 6 and 7, we obtain that $\|\Lambda(t,s)\| = \sup_{v,u\in\mathbb{C}^{2n}} |v^\dagger\Lambda(t,s)u|/\|v\|\|u\| \leq 1$.

Let us now consider bounding $Z(t)$ defined in lemma 3. We will provide a detailed analysis for an upper bound on one of the four terms appearing in $Z(t)$ — the other terms can be bounded similarly. Consider the first term — we have that

$$\left\|\int_0^t \tilde{L}^*(s-t)\Lambda^{\mathrm{T}}(t,s)ds\right\| = \sup_{\substack{v,u\in\mathbb{C}^{2n}\\ \|v\|,\|u\|=1}} \left|\int_0^t v^\dagger\tilde{L}^*(s-t)\Lambda^{\mathrm{T}}(t,s)u ds\right| = \left|\sup_{\substack{v,u\in\mathbb{C}^{2n}\\ \|v\|,\|u\|=1}} \int_0^t v^\dagger\tilde{L}^*(s-t)w(s)ds\right|,$$

where $w(s) = \Lambda^{\mathrm{T}}(t,s)u$. Now, by the definition of $K(s)$, we obtain

$$\left|\int_0^t v^\dagger \tilde{L}^*(s-t)w(s)ds\right| = \left|\int_0^t \sum_{j,z}\sum_{x,\alpha}\sum_{x',\alpha'} K^*_{j,z}(s-t)v_x^{\alpha^*} l^{\alpha^*}_{j,z;x} l^{\alpha'}_{j,z;x'} w^{\alpha'}_{x'}(s)ds\right|,$$
$$\leq \int_0^t K(s-t)\sum_{j,z}\left|\sum_{x,\alpha}\sum_{x',\alpha'} v_x^{\alpha^*} l^{\alpha^*}_{j,z;x} l^{\alpha'}_{j,z;x'} w^{\alpha'}_{x'}(s)\right| ds,$$
$$\leq \int_0^t K(s-t)|v|^{\mathrm{T}}\tilde{L}_m|w(s)|ds,$$

where $|v|$ and $|w(s)|$ are vectors formed by taking the absolute values of $v$ and $w(s)$ respectively, and $\tilde{L}_m \in \mathbb{R}^{2n\times 2n}$ is a matrix given by

$$\left(\tilde{L}_m\right)^{x',\alpha'}_{x,\alpha} = \sum_{j,z} |l^{\alpha}_{j,z;x}||l^{\alpha'}_{j,z;x'}|.$$

Now, from lemma 4, it follows that $\|\tilde{L}_m\| \leq \sqrt{2Dn_L}(2R+1)^d\delta$, and consequently $|v|^{\mathrm{T}}\tilde{L}_m|w(s)| \leq \sqrt{2Dn_L}(2R+1)^d\delta\|v\|\|w(s)\| \leq \sqrt{2Dn_L}(2R+1)^d\delta\|v\|\|u\|$, where we have used the previously shown fact of $\|\Lambda(t,s)\| \leq 1$. Thus, we have that

$$\left|\int_0^t v^\dagger \tilde{L}^*(t-s)w(s)ds\right| \leq \sqrt{2Dn_L}(2R+1)^d\delta\|v\|\|u\|\int_0^t K(s-t)ds \leq \sqrt{2Dn_L}(2R+1)^d\delta\|v\|\|u\|,$$

and therefore

$$\left\|\int_0^t \tilde{L}^*(t-s)\Lambda^{\mathrm{T}}(t,s)ds\right\| \leq \sqrt{2Dn_L}(2R+1)^d\delta.$$

Performing a similar analysis to the remaining three terms in $Z(t)$, we obtain that $\|Z(t)\| \leq 2\sqrt{2Dn_L}(2R+1)^d\delta$.

*Proof (of proposition 1).* In this proof, we will follow the notation introduced in lemma 3 and use unprimed matrices for the noiseless (target) problem, and primed matrices for the noisy problem. From lemma 4, it follows that

$$\|X - X'\| \leq \|\tilde{H} - \tilde{H}'\| + \|\tilde{Q} - \tilde{Q}'\| \leq \delta_X := 2D(2R+1)^d\delta + 4Dn_L(2R+1)^{2d}(2\delta+\delta^2),$$

and

$$\|Y - Y'\| \leq \|\tilde{Q} - \tilde{Q}'\| \leq \delta_Y := 4Dn_L(2R+1)^{2d}(2\delta+\delta^2).$$

In the absence of noise and errors, the correlation matrix $\Gamma(t)$ is governed by the differential equation

$$\frac{d}{dt}\Gamma(t) = X\Gamma + \Gamma X^{\mathrm{T}} + Y \implies \Gamma(t) = e^{Xt}\Gamma(0)e^{X^{\mathrm{T}}t} + \int_0^t e^{X(t-s)}Ye^{X^{\mathrm{T}}(t-s)}ds.$$

Note that we have set $Z(t) = 0$, since in the noiseless problem there is no decohering environment. In the presence of noise and errors, we instead obtain that

$$\frac{d}{dt}\Gamma'(t) = X'\Gamma'(t) + \Gamma'(t)X'^{\mathrm{T}} + Y' + Z'(t),$$

from which it follows that

$$\Gamma'(t) - \Gamma(t) = \int_0^t e^{X(t-s)}\Big((X'-X)\Gamma'(t) + \Gamma'(t)(X'-X)^{\mathrm{T}} + (Y'-Y) + Z'(s)\Big)e^{X^{\mathrm{T}}(t-s)}ds. \quad (8)$$

We have already established in lemma 5 that $\|Z'(t)\| \leq \delta_Z = 2\sqrt{2Dn_L}(2R+1)^d\delta$. Furthermore, we note that $\|e^{Xt}\| = \|e^{X^{\mathrm{T}}t}\| \leq \|e^{(X+X^{\mathrm{T}})t/2}\| \leq 1$ (theorem IX.3.1 of Ref. [1]), where we have used the fact that $X + X^{\mathrm{T}} \preceq 0$. Consequently, from Eq. 8, we have that

$$\|\Gamma'(t) - \Gamma(t)\| \leq t(2\delta_X + \delta_Y + \delta_Z) \leq O(\delta t).$$

From this bound, we can now conclude a bound on the error in a quadratic observables. Suppose $O$ is a quadratic observable specified by the coefficients $o^{\alpha,\beta}_{x,y}$ i.e. as per Eq. 1, $O$ is given by

$$O = \sum_{x,y\in\mathbb{Z}_L^d}\sum_{\alpha,\beta=1}^{2D} o^{\alpha,\beta}_{x,y} c^\alpha_x c^\beta_y.$$

Let $\mathcal{O}$ and $\mathcal{O}'$ be respectively the noisy and noiseless expected values of this observable. Then,

$$|\mathcal{O}-\mathcal{O}'| = |\text{Tr}\big(\tilde{O}(\Gamma(t)-\Gamma'(t))\big)| \leq \|\tilde{O}\|_{\text{op},1}\|\Gamma(t)-\Gamma'(t)\|,$$

where $\tilde{O}$ is the matrix of coefficients $o^{\alpha,\beta}_{x,y}$ with $(x,\alpha)$ corresponding to the row index and $(y,\beta)$ corresponding to the column index. Since we have already established $\|\Gamma(t)-\Gamma'(t)\| \leq O(\delta t)$, it only remains to prove that $\|\tilde{O}\|_{\text{op},1}$ is bounded above by a system-size independent constant. Observe that since $O$ only acts on $k$ sites, $\tilde{O}$ has at most $2kD$ nonzero eigenvalues, thus $\|\tilde{O}\|_{\text{op},1} \leq 2kD\|\tilde{O}\|$. Assuming the observable to be normalized such that $\|O\|\leq 1 \implies \|\tilde{O}\|\leq 1$, we thus obtain that $|\mathcal{O}-\mathcal{O}'| \leq O(\delta t)$.

### B. Proof of proposition 2 (Ground states of local Gaussian fermionic models)

In this appendix we will prove the stability of the expectation value of a translationally invariant, k-locally generated Gaussian observable on the ground state of a quadratic Hamiltonian. We first provide a lemma that uses the translation invariance of a local observable to provide an error bound.

*Lemma* 6. Consider a quadratic operator $O$ which is translationally invariant and expressible as

$$O = \frac{1}{n}\sum_{x\in\mathbb{Z}_L^d}\tau_x(O_0),$$

where $n = L^d$ is the number of sites in $\mathbb{Z}_L^d$, $O_0$ is a quadratic operator with a support on at most $k$ sites and $\tau_x$ is a super-operator that translates an operator by $x$, then for any quadratic operator $A_0$,

$$\big|\text{Tr}(O^\dagger A_0)\big| \leq \frac{4D^2k}{n}\|\tilde{O}_0\|\|\tilde{A}_0\|_{\text{op},1}.$$

*Proof.* We note that

$$\text{Tr}(\tilde{O}^\dagger\tilde{A}_0) = \frac{1}{n}\sum_{x\in\mathbb{Z}_L^d}\text{Tr}\big(\tilde{O}_0\tau_x^\dagger(\tilde{A}_0)\big) = \frac{1}{n}\sum_{x\in\mathbb{Z}^d}\text{Tr}\big(\tilde{O}_0\tau_{-x}(\tilde{A}_0)\big).$$

Define $A \equiv n^{-1}\sum_{x\in\mathbb{Z}_L^d}\tau_{-x}(A_0)$. We note that $A$ is translationally invariant on the underlying lattice — consequently, if $F$ is the $n\times n$ Fourier transform matrix, then $F_D = F\otimes I_{2D}$ block diagonalizes $\tilde{A}$ i.e. $F_D\tilde{A}F_D^\dagger$ will be a block diagonal matrix with $n$ blocks of size $2D\times 2D$. Then we use

$$\Big|\text{Tr}(\tilde{O}^\dagger\tilde{A}_0)\Big| = \Big|\text{Tr}(F_D^\dagger\tilde{O}^\dagger F_D F_D^\dagger\tilde{A}F_D)\Big| \leq \Big\|\text{vec}\big(F_D^\dagger\tilde{O}_0F_D\big)\Big\|_\infty\Big\|\text{vec}(F_D^\dagger\tilde{A}F_D)\Big\|_1$$

where we have applied Hölder's inequality to the norms of the vectorized matrices. Now we bound each of the factors in the right hand side. Since the operator $O_0$ has support only $k$ sites, $\tilde{O}_0$ only has $2Dk\times 2Dk$ non-zero elements. Suppose that $\Pi_{O_0}$ is a diagonal matrix with 1s on the entries that correspond to non-zero elements of $\tilde{O}_0$ — it then follows that $\tilde{O}_0 = \Pi_{O_0}\tilde{O}_0\Pi_{O_0}$. We further note that if $f_i$ is the $i^{\text{th}}$ column of $F_D$ then

$$\Big\|\text{vec}\big(F_D^\dagger\tilde{O}_0F_D\big)\Big\|_\infty = \sup_{i,j}\Big|f_i^\dagger\Pi_{O_0}\tilde{O}_0\Pi_{O_0}f_j\Big| \leq \|\tilde{O}_0\|\sup_{i,j}\|\Pi_{O_0}f_i\|\|\Pi_{O_0}f_j\| = \frac{2Dk}{n}\|\tilde{O}_0\|,$$

where we have used that each entry of $F_D$ has magnitude $1/\sqrt{n}$ since it is the Fourier transform matrix. Next, since $F_D^\dagger\tilde{A}F_D$ is block diagonal with $N$ $2D\times 2D$ blocks, and labelling by $A_1, A_2\dots A_N$ these blocks, we obtain that

$$\Big\|\text{vec}(F_D^\dagger\tilde{A}F_D)\Big\|_1 = \sum_{i=1}^N\|\text{vec}(A_i)\|_1 \leq 2D\sum_{i=1}^N\|A_i\|_{\text{op},1} = 2D\Big\|F_D^\dagger\tilde{A}F_D\Big\|_{\text{op},1} = 2D\Big\|\tilde{A}\Big\|_{\text{op},1}.$$

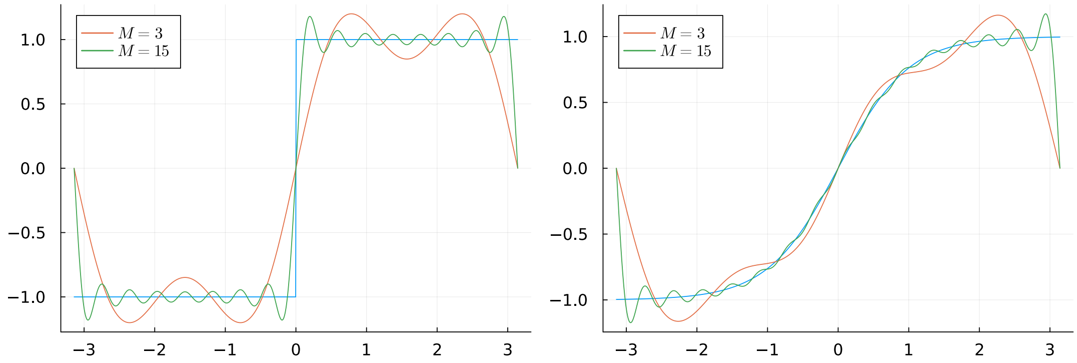


Supplemental Figure 1. (left) Truncated Fourier series approximation $\mathrm{sign}_M(x)$ to the $\mathrm{sign}(x)$ function, used in the proof of proposition 2. (right) Truncated Fourier series approximation $t_M(x)$ to the $\tanh(\beta x)$ function (for $\beta = 1$), used in the proof of proposition 3.

where we have used $\|\mathrm{vec}(M)\|_1 \leq n\|M\|_{\mathrm{op},1}$ for an $n \times n$ matrix[1]. Finally, since $A = \sum_{x \in \mathbb{Z}_L^d} \tau_{-x}(A_0)/N$, it follows that $\|\tilde{A}\|_{\mathrm{op},1} \leq \|\tilde{A}_0\|_{\mathrm{op},1}$. Combining the above estimates, the lemma statement follows.

The correlation matrix $\Gamma$ of the ground state of a quadratic Hamiltonian $H$ with matrix of coefficients $\tilde{H}$ (see Eq. (1)) is given by

$$\Gamma = \mathrm{sign}(\tilde{H}),$$

where $\mathrm{sign}(x) = x/|x|$ for $x \neq 0$ and 0 for $x = 0$[2]. The sign function applied on a matrix is to be understood as an operator function i.e. as a function acting on the eigenvalues of the argument while keeping the eigenvectors unchanged. Our proof will rely on a Fourier series approximation to the sign function. Within the interval $(-\pi, \pi)$, we will investigate the approximation of $\mathrm{sign}(x)$ with $\mathrm{sign}_M(x)$, where

$$\mathrm{sign}_M(x) = \sum_{n=-M}^{M} c_n e^{inx} \text{ where } c_n = \frac{1}{2\pi}\int_{-\pi}^{\pi} \mathrm{sign}(x) e^{-inx} dx.$$

To analyze the error between $\mathrm{sign}_M(x)$ and $\mathrm{sign}(x)$, it is convenient to express $\mathrm{sign}_M(x)$ in terms of the Dirichlet kernel,

$$\mathrm{sign}_M(x) \equiv \int_{-\pi}^{\pi} D_M(x-y)\mathrm{sign}(y) dy,$$

where

$$D_M(x) \equiv \frac{1}{2\pi}\sum_{n=-M}^{M} e^{-inx} = \frac{1}{2\pi}\frac{\sin[(M+1/2)x]}{\sin(x/2)}.$$

Below, we provide two technical lemmas about the $\mathrm{sign}_M$ function — one that quantifies the approximation error between it and the exact sign function, and the next that quantifies the maximum value of the $\mathrm{sign}_M$ function. Both of these lemmas will be used for the perturbation theory analysis of the free-fermion ground state problem.

*Lemma 7.* For all $\eta \leq |x| \leq \pi - \eta$ and $M > 0$,

$$\left|\mathrm{sign}(x) - \mathrm{sign}_M(x)\right| \leq \frac{1}{M} + \frac{1}{M\eta}.$$

---

[1] To see this, let $\sigma_{ij} \equiv \mathrm{sign}(M_{ji})$. Then $\|\sigma\|_{\mathrm{op}} \leq n\|\mathrm{vec}(\sigma)\|_\infty = n$, and $\|\mathrm{vec}(M)\|_1 = \mathrm{Tr}(\sigma M) \leq \|\sigma\|_{\mathrm{op}}\|M\|_{\mathrm{op},1} \leq n\|M\|_{\mathrm{op},1}$.

[2] The reader may be familiar with the equivalent formulation in terms of complex fermions, where the function to be applied to the Hamiltonian matrix to obtain the correlation matrix of the ground state is of the Heaviside type, such that it populates negative energy states and depopulates positive energy states. The function of the sign function in the language of Majorana fermions is exactly analogous.

*Proof.* We first consider $x \in [\eta, \pi]$. We note that

$$\operatorname{sign}_M(x) = \int_0^\pi D_M(x-y)dy - \int_0^\pi D_M(x+y)dy.$$

Now, since $\int_{-\pi}^{\pi} D_M(y)dy = 1$, we obtain that

$$\int_0^\pi D_M(x-y)dy = 1 - \int_0^\pi D_M(x+y)dy,$$

and thus

$$|\operatorname{sign}_M(x) - \operatorname{sign}(x)| = 2\left|\int_0^\pi D_M(x+y)dy\right|.$$

Next, we apply integration by parts to obtain

$$\int_0^\pi D_M(x+y)dy = \frac{1}{\pi(2M+1)}\Bigg(\frac{\cos((M+1/2)(\pi+x))}{\cos(x/2)} + \frac{\cos((M+1/2)(\pi+x))}{\sin(x/2)} - \frac{1}{2}\int_0^\pi \frac{\cos((M+1/2)(x+y))\cos((x+y)/2)}{\sin^2((x+y)/2)}dy\Bigg),$$

and therefore

$$\begin{aligned}\left|\int_0^\pi D_M(x+y)dy\right| &\leq \frac{1}{\pi(2M+1)}\left(\frac{1}{|\cos(x/2)|} + \frac{1}{|\sin(x/2)|} + \frac{1}{2}\int_0^\pi \frac{|\cos((x+y)/2)|dy}{\sin^2((x+y)/2)}\right) \\ &\leq \frac{2}{\pi(2M+1)}\left(\frac{1}{|\cos(x/2)|} + \frac{1}{|\sin(x/2)|} - 1\right),\end{aligned}$$

where in the last step we have used the integral

$$\frac{1}{2}\int_x^{\pi+x} \frac{|\cos(y/2)|}{\sin^2(y/2)}dy = \frac{1}{\sin(x/2)} + \frac{1}{\cos(x/2)} - 2.$$

Now, for $x \in (\eta, \pi/2)$, $|\cos(x/2)| \geq 1/\sqrt{2}$ and $|\sin(x/2)| \geq x/\pi \geq \eta/\pi$. Therefore, we obtain that

$$\left|\int_0^\pi D_M(x+y)dy\right| \leq \frac{2}{\pi(2M+1)}\left(\sqrt{2} + \frac{\pi}{\eta} - 1\right).$$

While this bound is true for $x \in [\eta, \pi/2]$, we note that both $\operatorname{sign}, \operatorname{sign}_M$ satisfy $f(x) = f(\pi - x)$ for $x \in [0, \pi]$ and consequently this bound also holds for $x \in [\pi/2, \pi - \eta]$. Finally, since for both $\operatorname{sign}, \operatorname{sign}_M$, $f(x) = -f(-x)$, it follows that this bound holds for $[-\pi + \eta, -\eta] \cup [\eta, \pi - \eta]$.

*Lemma* 8. For all $x \in [-\pi, \pi]$, $|\operatorname{sign}_M(x)| \leq 5$.

*Proof.* This proof is an adaptation of the standard technique based on Riemann integration that is used to treat Gibbs phenomena in Fourier analysis. We repurpose that technique to provide error bounds as a function of $M$ instead of just concentrating on the asymptotic limit $M \to \infty$. Again, we only consider $x \in [0, \pi/2]$, and extend the bound on $|\operatorname{sign}_M(x)|$ to the remaining interval by symmetry. We divide the interval $[0, \pi/2]$ into $[0, \alpha_0/M] \cup [\alpha_0/M, \pi/2]$, where $\alpha_0$ is a constant that we pick later.

Consider first $x \in [\alpha_0/M, \pi/2]$. An application of lemma 7 yields

$$|\operatorname{sign}_M(x)| \leq 1 + \frac{2}{\pi(2M+1)}\left(\sqrt{2} - 1 + \frac{\pi M}{\alpha_0}\right).$$

For large $M$, this bound scales as $\sim 1/\alpha_0$ and thus does not allow us to provide an upper bound on $\operatorname{sign}_M(x)$ for $x$ close to 0. For this, we use the representation of $\operatorname{sign}_M(x)$ as a Fourier series which approximates a Riemann integral of $\sin(t)/t$. Consider $x \in [0, \alpha_0/M)$ and let $\alpha = xM$ ($\alpha \leq \alpha_0$). Note that

$$\operatorname{sign}_M(x) = \frac{2}{\pi}\sum_{k \in [1,M] | k \text{ is odd}} \frac{2}{k}\sin\left(\frac{k\alpha}{M}\right)$$

To bound the term in the summation, we observe that it is an approximation of the Riemann integral of $\sin(\alpha x)/x$ in the interval $[0,1]$. In particular, since $\sup_{x\in\mathbb{R}} |(\sin x/x)'| \leq 2$, Taylor's theorem yields that

$$\left| \sum_{k\in[1,M] | k \text{ is odd}} \frac{2}{k} \sin\left(\frac{k\alpha}{M}\right) - \int_0^1 \frac{\sin \alpha x}{x} dx \right| \leq \frac{4\alpha^2}{M} \leq \frac{4\alpha_0^2}{M}.$$

Finally, we note that

$$\int_0^1 \frac{\sin \alpha x}{x} dx \leq \alpha \leq \alpha_0.$$

Thus, we obtain that for $x \in [0, \alpha_0/M)$,

$$|\text{sign}_M(x)| \leq \alpha_0 + \frac{4\alpha_0^2}{M}.$$

Thus, for the entire interval $[0, \pi/2]$, we obtain that

$$|\text{sign}_M(x)| \leq \max\left(\alpha_0 + \frac{4\alpha_0^2}{M}, 1 + \frac{2}{\pi(2M+1)}\left(\sqrt{2} - 1 + \frac{\pi M}{\alpha_0}\right)\right).$$

Since this holds for any $\alpha_0$, we choose $\alpha_0 = 1$. We then obtain that

$$|\text{sign}_M(x)| \leq \max\left(1 + \frac{4}{M}, 1 + \frac{2}{\pi(2M+1)}\big(\sqrt{2} - 1 + \pi M\big)\right) \leq 5 \text{ for } M \geq 1.$$

*Proof (of proposition 2).* The expectation value of the observable $O$ in the ground state of the Hamiltonian $H$ is given by

$$\langle O\rangle_H = \text{Tr}\big(\tilde{O}\ \text{sign}(\tilde{H})\big),\ \langle O\rangle_{H'} = \text{Tr}\big(\tilde{O}\ \text{sign}(\tilde{H}')\big).$$

Without loss of generality, we will assume that $\tilde{H}, \tilde{H}'$ are normalized so that $\|\tilde{H}\|, \|\tilde{H}'\| \leq \frac{\pi}{2}$. This way all the eigenfrequencies lie in the interval $[-\frac{\pi}{2}, \frac{\pi}{2}]$. Lemma 1 guarantees that this can be done with a constant normalization factor, i.e. one that does not depend on the system size, and does not change the ground state (note however that $\delta$ and $f_h$ would have to be rescaled accordingly). Now, from lemma 6, it follows that

$$|\langle O\rangle_H - \langle O\rangle_{H'}| \leq \frac{4D^2k}{n}\|\tilde{O}_0\| \|\text{sign}(\tilde{H}) - \text{sign}(\tilde{H}')\|_{\text{op},1}.$$

Furthermore,

$$\begin{aligned}&\|\text{sign}(\tilde{H}) - \text{sign}(\tilde{H}')\|_{\text{op},1} \leq \\ &\qquad \|\text{sign}(\tilde{H}) - \text{sign}_M(\tilde{H})\|_{\text{op},1} + \|\text{sign}(\tilde{H}') - \text{sign}_M(\tilde{H}')\|_{\text{op},1} + \|\text{sign}_M(\tilde{H}) - \text{sign}_M(\tilde{H}')\|_{\text{op},1}.\end{aligned}$$

We bound each term on the right hand side separately. Consider $\|\text{sign}(\tilde{H}) - \text{sign}_M(\tilde{H})\|_{\text{op},1}$ — denoting by $\lambda_i$ the eigenvalues of $\tilde{H}$ and for any $\eta > 0$, we can express it as

$$\|\text{sign}(\tilde{H}) - \text{sign}_M(\tilde{H})\|_{\text{op},1} = \sum_{i|\lambda_i\in[-\eta,\eta]} |\text{sign}(\lambda_i) - \text{sign}_M(\lambda_i)| + \sum_{i|\lambda_i\notin[-\eta,\eta]} |\text{sign}(\lambda_i) - \text{sign}_M(\lambda_i)|.$$

The motivation behind splitting the error into these two terms is that, within the interval $[-\frac{\pi}{2}, \frac{\pi}{2}]$, the approximation of $\text{sign}\,(\lambda)$ by $\text{sign}_M\,(\lambda)$ is only good outside the neighbourhood of 0 (see Fig. 1) — consequently, we treat the eigenvalues of $\tilde{H}$ which lie within $\eta$ radius of 0 separately from the rest. It now follows that from assumption **??** and lemma 8 that

$$\sum_{i|\lambda_i\in[-\eta,\eta]} |\text{sign}(\lambda_i) - \text{sign}_M(\lambda_i)| \leq 6nf_h(\eta) + 6\kappa(\eta, n).$$

Furthermore, from lemma 7,

$$\sum_{i|\lambda_i\notin[-\eta,\eta]} |\text{sign}(\lambda_i) - \text{sign}_M(\lambda_i)| \leq \frac{n}{M}\left(1+\frac{1}{\eta}\right).$$

Therefore, we obtain that

$$\frac{1}{n}\|\text{sign}(\tilde{H}) - \text{sign}_M(\tilde{H})\|_{\text{op},1} \leq 6f_h(\eta) + 6\frac{\kappa(\eta,n)}{n} + \frac{1}{M}\left(1+\frac{1}{\eta}\right).$$

We can similarly analyze $\|\text{sign}(\tilde{H}') - \text{sign}(\tilde{H}')\|_{\text{op},1}$. Denote by $\lambda_i'$ the eigenvalues of $\tilde{H}'$ — it follows from Weyl's theorem and lemma 4 that $|\lambda_i - \lambda_i'| \leq \|\tilde{H} - \tilde{H}'\|_{\text{op}} \leq c_0\delta$ where $c_0 = 2D(2R+1)^d$. Consequently, for sufficiently small, but $\Theta(1)$, $\delta$, we obtain that

$$\sum_{i|\lambda_i'\in[-\eta,\eta]} |\text{sign}(\lambda_i') - \text{sign}_M(\lambda_i')| \leq 6nf_h(\eta+c_0\delta) + 6\kappa(\eta+c_0\delta,n),$$

and

$$\sum_{i|\lambda_i'\notin[-\eta,\eta]} |\text{sign}(\lambda_i') - \text{sign}_M(\lambda_i')| \leq \frac{n}{M}\left(1+\frac{1}{\eta}\right).$$

Therefore,

$$\frac{1}{n}\|\text{sign}(\tilde{H}') - \text{sign}_M(\tilde{H}')\|_{\text{op},1} \leq 6f_h(\eta+c_0\delta) + 6\frac{\kappa(\eta+c_0\delta,n)}{n} + \frac{1}{M}\left(1+\frac{1}{\eta}\right).$$

Finally, we consider $\|\text{sign}_M(H) - \text{sign}_M(H')\|_{\text{op},1} \leq n\|\text{sign}_M(H) - \text{sign}_M(H')\|$. Now, denoting by $\{c_m\}_{m\in\mathbb{Z}}$ the Fourier series components of sign function, then

$$\|\text{sign}_M(H) - \text{sign}_M(H')\| \leq \sum_{m=-M}^{M} |c_m|\|e^{im\tilde{H}} - e^{im\tilde{H}'}\| \leq \sum_{m=-M}^{M} |mc_m|\|\tilde{H} - \tilde{H}'\|.$$

Using the explicit expression for $c_m$, we can immediately conclude that $|mc_m| = 2/\pi$ when $m$ is odd, and 0 when $m$ is even. Therefore, we obtain that

$$\|\text{sign}_M(H) - \text{sign}_M(H')\| \leq \frac{2(M+1)}{\pi}\|\tilde{H} - \tilde{H}'\| \leq \frac{2(M+1)}{\pi}c_0\delta.$$

Combining all of these estimates, we obtain that

$$\frac{1}{n}\|\text{sign}(\tilde{H}) - \text{sign}(\tilde{H}')\|_{\text{op},1} \leq \frac{2(M+1)}{\pi}c_0\delta + \frac{2}{M}\left(1+\frac{1}{\eta}\right) + 6\big(f_h(\eta) + f_h(\eta+c_0\delta)\big) + 6\left(\frac{\kappa(\eta,n)}{n} + \frac{\kappa(\eta+c_0\delta,n)}{n}\right).$$

with $c, c'$ constants. Since this is valid for any $\eta$ and $M$, choosing $M = \delta^{-1/2}$ and $\eta = \delta^{1/4}$, we obtain the proposition.

### C. Proof of proposition 3 (Gibbs state of Gaussian fermion models)

The correlation matrix of a thermal state of a quadratic Hamiltonian can be written in terms of the coefficient matrix $H$ of the latter as $\Gamma = \tanh(\beta H)$. Note that the $\beta \to \infty$ limit yields the sign function, which was used in the previous appendix to compute the ground state correlation matrix. Indeed, the reasoning here will be similar to that of appendix II B, replacing the sign function with the hyperbolic tangent. The next couple of lemmas discuss the Fourier series approximation of $\tanh\beta x$, defined as

$$t_M(x) \equiv \sum_{n=-M}^{M} c_n e^{inx}, \text{ where } c_n = \frac{1}{2\pi}\int_{-\pi}^{\pi} \tanh\beta x e^{-inx}dx.$$

*Lemma* 9. For $M \geq 1$, and $x \in \left[-\frac{\pi}{2}, \frac{\pi}{2}\right]$,

$$|t_M(x) - \tanh \beta x| \leq \frac{q(\beta)}{M},$$

where $q(\beta) \equiv 12\pi^2\beta^3 + 2\pi^2\beta^2 + \left(2 + \frac{\pi^2}{2}\right)\beta + \left(\frac{4\sqrt{2}}{\pi} + \frac{\pi^2}{2}\right) = O(\beta^3)$.

*Proof.* We fix the value of $\beta$ and let $t(x)$ be the $2\pi$-periodic extension of $\tanh \beta x$ i.e.

$$t(x) \equiv \tanh \beta(x - 2n\pi), \qquad x - 2n\pi \in [-\pi, \pi], \quad n \in \mathbb{Z}. \tag{9}$$

We use this $2\pi$-periodic extension of $\tanh \beta x$ since it will allow us to represent and approximate $\tanh \beta x$ as a truncated fourier series, which would then allow us to use the results from stability of dynamics in understanding stability of Gibb's state. Once again, it will be convenient to represent $t_M(x)$ in terms of the Dirichlet kernel $D_M$. We note,

$$t_M(x) = \int_{-\pi}^{\pi} D_M(x-y)t(y)dy = \int_{-\pi}^{\pi} D_M(y)t(x-y)dy = \int_{-\pi}^{\pi} D_M(y)t(x+y)dy,$$

where in the last step we have used the fact that $D_M$ is an even function (i.e. $D_M(y) = D_M(-y)$). Therefore, using that the Dirichlet kernel is normalized, we write

$$t(x) - t_M(x) = \frac{1}{2}\int_{-\pi}^{\pi} D_M(y)\left(2t(x) - t(x-y) - t(x+y)\right)dy = \int_0^{\pi} D_M(y)f_x(y)dy,$$

where in the last step we have defined $f_x(y) \equiv 2t(x) - t(x-y) - t(x+y)$. In the integration interval $[0, \pi]$, $f_x(y)$ is piecewise smooth with a single jump discontinuity at $y = \pi - x$. We thus split the integral into the two intervals $[0, \pi - x]$ and $[\pi - x, \pi]$ and apply integration by parts in each of them. For the first one,

$$\int_0^{\pi-x} D_M(y)f_x(y)dy = -\frac{1}{\pi}\frac{\cos\left(\left(M+\frac{1}{2}\right)y\right)}{2M+1}\frac{f_x(y)}{\sin\frac{y}{2}}\Bigg|_{y=0}^{\pi-x} + \frac{1}{(2M+1)\pi}\int_0^{\pi-x} g_x(y)\frac{\cos\left(\left(M+\frac{1}{2}\right)y\right)}{\sin^2\frac{y}{2}}dy$$

where $g_x(y) \equiv 2\sin\frac{y}{2}f_x'(y) - \cos\frac{y}{2}f_x(y)$. To bound this expression, we will use the following properties of the functions $f_x(y), g_x(y)$ on the interval $[0, \pi - x]$, where they are smooth:

$$f_x(0) = f_x'(0) = 0, |f_x(y)| \leq 4, |f_x'(y)| \leq 2\beta, |f_x''(y)| \leq 4\beta^2, |f_x'''(y)| \leq 12\beta^3,$$
$$g_x(0) = g_x'(0) = 0, |g_x''(y)| \leq 24\beta^3 + 4\beta^2 + \beta + 1.$$

These bounds follow from direct computation, and in the case of $g_x(y)$ they are easiest to see when expressed in terms of $f_x(y)$. They imply (via Taylor's theorem with second order remainder) that

$$|g_x(y)| \leq (24\beta^3 + 4\beta^2 + \beta + 1)\frac{y^2}{2}$$

which together with $\sin^2(y) \geq \frac{y^2}{\pi^2}$ will allow us to bound the integral. Putting it all together, we have

$$\left|\int_0^{\pi-x} D_M(y)f_x(y)dy\right| \leq \frac{4\sqrt{2}}{(2M+1)\pi} + \frac{\pi^2}{(2M+1)}(24\beta^3 + 4\beta^2 + \beta + 1)$$

Now we proceed on to the second interval $y \in [\pi - x, \pi]$ and similarly integrate by parts,

$$\int_{\pi-x}^{\pi} D_M(y)f_x(y)dy = -\frac{1}{\pi}\frac{\cos\left(\left(M+\frac{1}{2}\right)y\right)}{2M+1}\frac{f_x(y)}{\sin\frac{y}{2}}\Bigg|_{y=\pi-x}^{\pi} + \frac{1}{(2M+1)\pi}\int_{\pi-x}^{\pi} g_x(y)\frac{\cos\left(\left(M+\frac{1}{2}\right)y\right)}{\sin^2\frac{y}{2}}dy.$$

Now the bound on $g_x(y)$ from Taylor's theorem no longer holds, due to the discontinuity, but since $y = 0$ is not in the integration interval, we can just use the constant bound $|g(x)| \leq 4\beta + 4$ to obtain

$$\left|\int_{\pi-x}^{\pi} D_M(y)f_x(y)dy\right| \leq \frac{4\sqrt{2}}{(2M+1)\pi} + \frac{4}{(2M+1)}(\beta + 1),$$

and putting everything together the lemma follows.

*Lemma* 10. If $\{c_n\}_{n\in\mathbb{Z}}$ are the Fourier series coefficients of $\tanh \beta x$ in the interval $[-\pi, \pi]$, then for $M \geq 1$

$$\sum_{n=-M}^{M} |nc_n| \leq 2M(\beta + 1).$$

*Proof.* This follows by a straightforward manipulation of $c_n$ — note that $c_0 = 0$, and for $n \neq 0$, we obtain from integration by parts that

$$c_n = \frac{1}{2\pi}\int_{-\pi}^{\pi} \tanh \beta x \; e^{-inx} dx = \frac{1}{2\pi}\bigg(\frac{2i}{n}\tanh \beta\pi \; e^{-in\pi} + \frac{\beta}{in}\int_{-\pi}^{\pi}\frac{e^{-inx}}{\cosh^2 \beta x}dx\bigg).$$

Consequently,

$$|c_n| \leq \frac{1}{2\pi}\bigg(\frac{2}{n} + \frac{2\pi\beta}{n}\bigg) \leq \frac{\beta + 1}{n}.$$

From this bound, the lemma follows.

*Proof (of proposition 3).* We bound the error between $\langle O\rangle_{H,\beta}$ and $\langle O\rangle_{H',\beta}$ using the same procedure as for the ground state (see appendix II B) — the proof simplifies significantly because $\tanh \beta x$ does not have a discontinuity near $x = 0$ (unlike the sign function). From lemma 6 it follows that

$$|\langle O\rangle_{H,\beta} - \langle O\rangle_{H',\beta}| \leq \frac{4D^2k}{n}\|\tilde{O}_0\|\|\tanh \beta\tilde{H} - \tanh \beta\tilde{H}'\|_{\text{op},1}.$$

We again split

$$\|\tanh \beta\tilde{H} - \tanh \beta\tilde{H}'\|_{\text{op},1} \leq \\ \|\tanh \beta\tilde{H} - t_M(\tilde{H})\|_{\text{op},1} + \|\tanh \beta\tilde{H}' - t_M(\tilde{H}')\|_{\text{op},1} + \|t_M(\tilde{H}') - t_M(\tilde{H})\|_{\text{op},1}$$

We will assume once again that $\|H\|, \|H'\| \leq \frac{\pi}{2}$, so that from lemma 9, it follows that

$$\|\tanh \beta\tilde{H} - t_M(\tilde{H})\|_{\text{op},1}, \|\tanh \beta\tilde{H}' - t_M(\tilde{H}')\|_{\text{op},1} \leq \frac{nq(\beta)}{M},$$

and

$$\|t_M(\tilde{H}) - t_M(\tilde{H}')\|_{\text{op},1} \leq n\|t_M(\tilde{H}) - t_M(\tilde{H}')\|_{\text{op}} \leq n\sum_{m=-M}^{M}|c_m|\|e^{im\tilde{H}} - e^{im\tilde{H}'}\|_{\text{op}}.$$

Furthermore, from lemmas 1, 2 and 4 we have $\|e^{im\tilde{H}} - e^{im\tilde{H}'}\|_{\text{op}} \leq mc_0\delta$, where $c = 2D(2R+1)^d$. Thus, from lemma 10, it follows that

$$\|t_M(\tilde{H}) - t_M(\tilde{H}')\|_{\text{op},1} \leq 2nM(\beta + 1)c_0\delta.$$

Thus, we obtain that for any $M > 1$,

$$|\langle O\rangle_{H,\beta} - \langle O\rangle_{H',\beta}| \leq 4D^2k\|O_0\|_{\text{op}}\bigg(\frac{2q(\beta)}{M} + 2(\beta + 1)c_0M\delta\bigg).$$

choosing $M = \sqrt{q(\beta)/c_0(\beta + 1)\delta}$, we obtain the result.

### D. Proof of proposition 4 (Fixed points of Gaussian fermiion models)

We will now consider translationally invariant local observables in the fixed point. Recall from section II A, lemma 3 that the dynamics of the correlation matrix $\Gamma(t)$ under evolution by a Gaussian master equation is governed by

$$\frac{d}{dt}\Gamma(t) = X\Gamma(t) + \Gamma(t)X^{\text{T}} + Y,$$

where $X, Y$ are defined in lemma 3. Assuming $X$ to be invertible, this differential equation has a unique fixed point which can be expressed as

$$\Gamma_\infty = \int_0^\infty e^{Xt} Y e^{X^{\mathrm{T}} t} dt.$$

We also note from lemma 3 is $X + X^{\mathrm{T}}$ is a negative definite matrix if $X$ is invertible, and its eigenvalues can be interpreted as a measure of the decay rates of the eigenmodes of the open system. We now restate assumption 2 in terms of the eigenvalues of $X + X^{\mathrm{T}}$.

*Assumption* 2 (*repeated*) The matrix $X + X^{\mathrm{T}}$ has no zero eigenvalues. Furthermore, if its eigenvalues are $-\lambda_i$, for $i \in \{1, 2 \dots 2n\}$ where $0 < \lambda_1 \leq \lambda_2 \dots \lambda_N$, then the number of eigenvalues in the interval $(0, \eta]$, $n(\eta)$ satisfies

$$n(\eta) \leq n f_\ell(\eta) + \kappa(\eta, n),$$

where $f_\ell$ is a function such that $f_\ell(\eta) \leq O(\eta^\alpha)$ for some $\alpha > 0$ and $\kappa(\eta, n) = o(n)$ for any fixed $\eta$.

This assumption is expected to be satisfied for translationally invariant systems, as well as rapidly mixing systems where typically there would be a gap in the Lindbladian spectrum. However, it additionally includes systems where the minimum eigenvalue could have a real part that scales as $1/n$, and thus would generically need $\Theta(n)$ time to reach their fixed points.

Similar to the stability result for ground states, the observables we will consider will be translationally invariant local observables - let $O_0$ be a $k$-local observable , then we consider observables $O$ which can be expressed as $O = \sum_{x \in \mathbb{Z}_L^d} \tau_x(O_0)/n$. We will denote by $\mathcal{O}$ and $\mathcal{O}'$ the expectation value of the observable $O$ in the unperturbed and perturbed fixed point i.e. $\mathcal{O} = \mathrm{Tr}(O\Gamma_\infty)$ and $\mathcal{O}' = \mathrm{Tr}(O\Gamma'_\infty)$.

*Proof (of proposition 4).* We start by using lemma 6 to obtain

$$|\mathcal{O} - \mathcal{O}'| \leq \frac{4D^2 k}{n} \|\tilde{O}_0\|_{\mathrm{op}} \|\Gamma_\infty - \Gamma'_\infty\|_{\mathrm{op},1}. \tag{10}$$

Furthermore,

$$\Gamma_\infty = \int_0^\infty e^{Xt} Y e^{X^{\mathrm{T}} t} dt \text{ and } \Gamma'_\infty = \int_0^\infty e^{X' t} Y' e^{X'^{\mathrm{T}} t} dt.$$

For any $t_0 > 0$, it follows that

$$\|\Gamma_\infty - \Gamma'_\infty\|_{\mathrm{op},1} \leq \frac{1}{n} \left\| \int_0^{t_0} \left( e^{Xt} Y e^{X^{\mathrm{T}} t} - e^{X' t} Y' e^{X'^{\mathrm{T}} t} \right) dt \right\|_{\mathrm{op},1} + \frac{1}{n} \left\| \int_{t_0}^\infty e^{Xt} Y e^{X^{\mathrm{T}} t} dt \right\|_{\mathrm{op},1} + \frac{1}{n} \left\| \int_{t_0}^\infty e^{X' t} Y' e^{X'^{\mathrm{T}} t} dt \right\|_{\mathrm{op},1}. \tag{11}$$

Furthermore, we note that

$$\int_{t_0}^\infty e^{Xt}(Y) e^{X^{\mathrm{T}} t} dt = e^{X t_0} \Gamma_\infty e^{X^{\mathrm{T}} t_0}.$$

Let us now estimate $\|e^{X t_0} \Gamma_\infty e^{X^{\mathrm{T}} t_0}\|_{\mathrm{op},1}$. Note that since $\Gamma_\infty$ is a covariance matrix, it is positive semi-definite and satifies $\|\Gamma_\infty\|_{\mathrm{op}} \leq 1$. Let $\Gamma_\infty = \sum_\alpha \sigma_\alpha |v_\alpha\rangle \langle v_\alpha|$ where $0 \leq \sigma_\alpha \leq 1$, then

$$e^{X t_0} \Gamma_\infty e^{X^{\mathrm{T}} t_0} = \sum_\alpha \sigma_\alpha e^{X t_0} |v_\alpha\rangle \langle v_\alpha| e^{X^{\mathrm{T}} t_0},$$

from which it follows that

$$\begin{aligned}
\|\Gamma_\infty\|_{\mathrm{op},1} &\leq \sum_\alpha \sigma_\alpha \|e^{X t_0} |v_\alpha\rangle\|^2, \\
&\leq \sum_\alpha \langle v_\alpha| e^{X^{\mathrm{T}} t_0} e^{X t_0} |v_\alpha\rangle, \\
&\leq \mathrm{Tr}(e^{X^{\mathrm{T}} t_0} e^{X t_0}) = \|e^{X t_0}\|_{\mathrm{op},2}^2, \\
&\leq \|e^{(X + X^T) t_0 / 2}\|_{\mathrm{op},2}^2.
\end{aligned}$$

Note that the first step follows from the variational definition of the Schatten-1 norm i.e. for any rank 1 decomposition of a matrix $A = \sum_\alpha c_\alpha |v_\alpha\rangle \langle u_\alpha|$ where $\|v_\alpha\|, \|u_\alpha\| = 1$, then $\|A\|_{\text{op},1} \leq \sum_\alpha |c_\alpha|$. The last step follows from theorem IX.3.1 of Ref. [1]. Next, $\|e^{(X+X^{\text{T}})t_0/2}\|_{\text{op},2}$ can be written explicitly in terms of the eigenvalues of $X + X^{\text{T}}$

$$\|e^{(X+X^T)t_0/2}\|_{\text{op},2}^2 = \sum_{i=1}^{2n} e^{-\lambda_i t_0} = \sum_{i|\lambda_i \in (0,\eta]} e^{-\lambda_i t_0} + \sum_{i|\lambda_i \in (\eta,\infty)} e^{-2\lambda_i t_0} \leq n\varphi(\eta) + 2ne^{-\eta t_0} + o(n)$$

Choosing $\eta = t_0^{-1+\beta}$ for any $\beta \in (0, 1)$, we obtain that $\|e^{(X+X^{\text{T}})t_0/2}\|_{\text{op},\,2} \leq n(f_\ell(t_0^{-1+\beta}) + 2e^{-t_0^\beta}) + o(n)$, which yields that

$$\frac{1}{n}\left\|\int_{t_0}^{\infty} e^{Xt} Y e^{X^{\text{T}}t} dt\right\|_{\text{op},1} \leq f_\ell(t_0^{-1+\beta}) + 2e^{-t_0^\beta} + o(1). \tag{12}$$

Following a similar procedure, we obtain that

$$\frac{1}{n}\left\|\int_{t_0}^{\infty} e^{X't} Y' e^{X'^{\text{T}}t} dt\right\|_{\text{op},1} \leq f_\ell(t_0^{-1+\beta} + c_0\delta) + e^{-t_0^\beta} + o(1), \tag{13}$$

where $c_0 = 4D(2R + 1)^d + 8Dn_L(2R + 1)^{2d}(2 + \delta) \leq O(1)$ is a constant that is independent of $n$, but dependent on $R, D, d, n_L$. In arriving at this result, we just need to account for the fact that the eigenvalue $\lambda_i'$ of $X' + X'^{\text{T}}$ could differ by at-most $2\|X - X'\|_{\text{op}}$ (which, from lemma 4, is $\leq c_0\delta$) from the corresponding eigenvalue $\lambda_i$ of $X + X^{\text{T}}$, and consequently the number of eigenvalues of $X' + X'^{\text{T}}$ in the interval $(0, \eta]$ is upper bounded by the number of eigenvalues of $X+X^{\text{T}}$ in the interval $(0, \eta+c_0\delta]$. In particular, this implies that $\sum_{i|\lambda_i' \in (0,\eta]} e^{-\lambda_i' t_0} \leq \sum_{i|\lambda_i \in (0,\eta+c_0\delta]} 1 \leq nf_\ell(\eta + c_0\delta) + o(n)$.

Let us now estimate the remaining term in $\|\Gamma_\infty - \Gamma_\infty'\|_{\text{op},1}$ — we bound the Schatten 1 norm by the operator (or Schatten $\infty$ norm) in the trivial way

$$\frac{1}{n}\left\|\int_0^{t_0} \left(e^{Xt} Y e^{X^{\text{T}}t} - e^{X't} Y' e^{X'^{\text{T}}t}\right) dt\right\|_{\text{op},1} \leq 2D \int_0^{t_0} \left\|e^{Xt} Y e^{X^{\text{T}}t} - e^{X't} Y' e^{X'^{\text{T}}t}\right\|_{\text{op}} dt. \tag{14}$$

Now, we can bound the error $\|e^{Xt} Y e^{X^{\text{T}}t} - e^{X't} Y' e^{X'^{\text{T}}t}\|_{\text{op}}$ using standard perturbation theory. We begin by noting from lemma 4 that $\|X - X'\|_{\text{op}}, \|Y - Y'\|_{\text{op}} \leq O(\delta)$ and $\|Y\|_{\text{op}}, \|Y'\|_{\text{op}} \leq O(1)$ . Furthermore, from theorem IX.3.1 of Ref. [1] and the fact that $X + X^{\text{T}}$, $X' + X'^{\text{T}}$ are negative-definite, $\|e^{Xt}\|_{\text{op}} \leq \|e^{(X+X^{\text{T}})t}\|_{\text{op}} \leq 1$ and $\|e^{X't}\|_{\text{op}} \leq \|e^{(X'+X'^{\text{T}})t}\|_{\text{op}} \leq 1$. Now, we note that

$$\|e^{Xt} Y e^{X^{\text{T}}t} - e^{X't} Y' e^{X'^{\text{T}}t}\|_{\text{op}} \leq \|e^{Xt}(Y - Y')e^{X^{\text{T}}t}\|_{\text{op}} + \|e^{Xt} Y' e^{X^{\text{T}}t} - e^{X't} Y' e^{X'^{\text{T}}t}\|_{\text{op}}.$$

We can bound both of these terms separately. For the first term, we obtain that

$$\|e^{Xt}(Y - Y')e^{X^{\text{T}}t}\|_{\text{op}} \leq \|e^{Xt}\|_{\text{op}} \|Y - Y'\|_{\text{op}} \|e^{X^{\text{T}}t}\|_{\text{op}} \leq \|Y - Y'\|_{\text{op}} \leq O(\delta).$$

For the second term, we obtain that

$$\begin{aligned}
&\|e^{Xt} Y' e^{X^{\text{T}}t} - e^{X't} Y' e^{X'^{\text{T}}t}\|_{\text{op}} \\
&\leq \int_0^t \left\|e^{X(t-s)}(X - X')e^{X's} Y' e^{X'^{\text{T}}s} e^{X^{\text{T}}(t-s)}\right\|_{\text{op}} ds + \int_0^t \left\|e^{X(t-s)} e^{X's} Y' e^{X'^{\text{T}}s}(X - X')^{\text{T}} e^{X^{\text{T}}(t-s)}\right\|_{\text{op}} ds, \\
&\leq 2\int_0^t \|e^{X(t-s)}\|_{\text{op}}^2 \|e^{X's}\|_{\text{op}}^2 \|X - X'\|_{\text{op}} \|Y\|_{\text{op}} ds \leq O(\delta t).
\end{aligned}$$

Thus, from Eq. 14, we obtain that

$$\frac{1}{n}\left\|\int_0^{t_0} \left(e^{Xt} Y e^{X^{\text{T}}t} - e^{X't} Y' e^{X'^{\text{T}}t}\right) dt\right\|_{\text{op},1} \leq O(t_0^2\delta) \tag{15}$$

Using the estimates in Eq. 10, 11, 12, 13 and 15, we have that for any $\beta \in (0, 1)$.

$$|\mathcal{O} - \mathcal{O}'| \leq O(\delta t_0^2) + f_\ell(t_0^{-1+\beta}) + f_\ell(t_0^{-1+\beta} + c_0\delta) + O(e^{-t_0^\beta}) + o(1).$$

Clearly, any choice of $t_0 = \delta^{-\alpha}$, where $\alpha < 1/2$ yields an upper bound on $|\mathcal{O} - \mathcal{O}'|$ that is uniform in $n$ and goes to 0 as $\delta \to 0$. A concrete choice could be $t_0 = \delta^{-1/4}$, and choosing $\beta \approx 0$, which yields $|\mathcal{O} - \mathcal{O}'| \leq O(\delta^{1/2}) + O(\varphi(\delta^{1/4}))$.

# Supplemental note III: Stability of spin models

## A. Proof of proposition 1 (Dynamics of locally interacting spin systems)

In this section, we will consider the target problem to be a spatially local Lindbladian

$$\mathcal{L} = \sum_{\alpha} \mathcal{L}_\alpha,$$

where $\|\mathcal{L}_\alpha\|_\diamond \leq 1$, and the support of $\mathcal{L}_\alpha$ denoted by $\Lambda_\alpha$ satisfies $\text{diam}(\Lambda_\alpha) \leq R \; \forall \alpha$. In the presence of coherent errors and incoherent noise, the quantum simulator instead implements a Lindbladian

$$\mathcal{L}'(t) = \sum_{\alpha} \mathcal{L}'_\alpha(t), \text{ where } \mathcal{L}'_\alpha(t) = \mathcal{L}'_\alpha - i\sum_{\alpha}[h_{\text{SE},\alpha}(t), \cdot] \text{ with } h_{\text{SE},\alpha}(t) = \sum_{j=1}^{n_L}\big(L_{j,\alpha}A^\dagger_{j,\alpha}(t) + L^\dagger_{j,\alpha}A_{j,\alpha}(t)\big). \tag{16}$$

Here $\mathcal{L}'_\alpha$ is the Linbladian implemented on qubits in $\Lambda_\alpha$ due to coherent errors, and we assume that $\|\mathcal{L}_\alpha - \mathcal{L}'_\alpha\| \leq \delta$. The Hamiltonian $h_{\text{SE},\alpha}(t)$ captures interaction of the qubits contained in $\Lambda_\alpha$ with an external decohering non-Markovian environment — the operators $A_{j,\alpha}(t)$ are assumed to be bosonic annihilation operators which satisfy $[A_{j,\alpha}(t), A^\dagger_{j',\alpha'}(t)] = \delta_{j,j'}\delta_{\alpha,\alpha'}K_{j,\alpha}(t)$ for a memory kernel $K_{j,\alpha}(t)$ and the operators $L_{j,\alpha}$ are system operators with support in $\Lambda_\alpha$ which also satisfy $\|L_{j,\alpha}\| \leq \sqrt{\delta}$. Similar to the noise model assumed for Gaussian fermion models, we assume that $K_{j,\alpha}(\tau)$ can have delta function contributions i.e.

$$K_{j,\alpha}(\tau) = K^c_{j,\alpha}(t) + \sum_{i=1}^{M} k^i_{j,\alpha}\delta(\tau - \tau_i),$$

where $K^c_{j,\alpha}(\tau)$ is a continuous function of $\tau$ and $k^i_{j,\alpha}$ are the amplitudes of the $\delta-$function contributions. We also assume that

$$\int_{\mathbb{R}} |K_{j,\alpha}(\tau)|d\tau \leq \int_{\mathbb{R}} |K^c_{j,\alpha}(\tau)|d\tau + \sum_{i=1}^{M}|k^i_{j,\alpha}| \leq 1. \tag{17}$$

This bound can be interpreted in a distributional sense by viewing $K_{j,\alpha}$ as a map that takes a continuous compact function $f$ and maps it to a complex number given by the integral $\int_{\mathbb{R}} K_{j,\alpha}(\tau)f(\tau)d\tau$. Equation 17 is then equivalent to requiring

$$\left|\int_{\tau_1}^{\tau_2} K_{j,\alpha}(\tau)f(\tau)d\tau\right| \leq \sup_{\tau\in[\tau_1,\tau_2]} |f(\tau)| \text{ for all continuous compact functions } f. \tag{18}$$

The main tool that we will use to prove the stability of local observables are the Lieb-Robinson bounds for spatially local Lindbladians.

*Lemma* 11 (Lieb-Robinson bounds, Ref. [2, 3]). Suppose $\mathcal{L} = \sum_\alpha \mathcal{L}_\alpha$ is a spatially local Lindbladian defined on a lattice $\mathbb{Z}^d_L$ such that $\|\mathcal{L}_\alpha\|_\diamond \leq 1$ and $\mathcal{L}_\alpha$ is supported on sites in $\Lambda_\alpha$ with $\text{diam}(\Lambda_\alpha) \leq R$. Suppose $O$ is an observable supported in $S_O \subseteq \mathbb{Z}^d_L$, and $\mathcal{K}_Y$ is a super-operator satisfying $\mathcal{K}_Y(I) = 0$ supported in $Y \subseteq \mathbb{Z}^d_L$, then $\exists \mu, v > 0$, independent of the system size $n = L^d$, such that

$$\|\mathcal{K}_Y(e^{\mathcal{L}^\dagger t}(O))\| \leq \|O\|\|\mathcal{K}_Y\|_{\infty\to\infty,cb} \min\big(|X|e^{-\mu d(S_O,Y)}(e^{vt}-1), 1\big).$$

We also provide another lemma which can be interpreted as a generalization of the input-output equations used in the theory of open quantum systems.

*Lemma* 12. Suppose $\mathcal{E}'(t,t') = \mathcal{T}\exp(\int_{t'}^{t}\mathcal{L}'(s)ds)$ is the channel generated by the Lindbladian $\mathcal{L}'(s)$ in Eq. 16. Then,

$$A_{j,\alpha}(t)\mathcal{E}'(t,0)(\cdot) = \mathcal{E}'(t,0)(A_{j,\alpha}(t)(\cdot)) - i\int_0^t K_{j,\alpha}(t-t')\mathcal{E}'(t,t')\big(L_{j,\alpha}\mathcal{E}'(t',0)(\cdot)]\big)dt'.$$

*Proof.* We will begin by defining, for $\varepsilon > 0$ and $n > m$, $\mathcal{D}'_\varepsilon[n,m]$ as

$$\mathcal{D}'_\varepsilon[n,m] = \prod_{k=n-1}^{m}\left(\text{id} + \int_{k\varepsilon}^{(k+1)\varepsilon}\mathcal{L}'(s)ds\right),$$

and noting that

$$\mathcal{E}'(t,s) = \lim_{\varepsilon\to 0} \mathcal{D}'_\varepsilon\left[\left\lceil \frac{t}{\varepsilon}\right\rceil, \left\lceil \frac{s}{\varepsilon}\right\rceil\right] \tag{19}$$

It is also convenient to define the superoperator $\mathcal{A}_{j,\alpha}(\tau)$ as the superoperator that left multiplies by $A_{j,\alpha}(\tau)$ i.e. $\mathcal{A}_{j,\alpha}(\tau)(X) = A_{j,\alpha}(\tau)X$. Now, from the explicit expression for $\mathcal{L}'(s)$, we can verify that for any operator $X$

$$\begin{aligned}
[\mathcal{A}_{j,\alpha}(\tau), \mathcal{L}'(s)](X) &= A_{j,\alpha}(\tau)\mathcal{L}'(s)(X) - \mathcal{L}'(s)(A_{j,\alpha}(\tau)X), \\
&= i\big(-A_{j,\alpha}(\tau)[h_{\mathrm{SE},\alpha}(s), X] + [h_{\mathrm{SE},\alpha}(s), A_{j,\alpha}(\tau)X]\big), \\
&= i(-[A_{j,\alpha}(\tau), h_{\mathrm{SE},\alpha}(s)]X \\
&= -iK_{j,\alpha}(\tau - s)L_{j,\alpha}X,
\end{aligned} \tag{20}$$

where we have used the fact that $[A_{j,\alpha}(\tau), h_{\mathrm{SE},\alpha}(s)] = [A_{j,\alpha}(\tau), A^\dagger_{j,\alpha}(s)L_{j,\alpha}] = [A_{j,\alpha}(\tau), A^\dagger_{j,\alpha}(s)]L_{j,\alpha} = K_{j,\alpha}(\tau-s)L_{j,\alpha}$. Next, note that

$$\mathcal{A}_{j,\alpha}(\tau)\mathcal{D}'_\varepsilon[n,0] = \mathcal{D}'_\varepsilon[n,0]\mathcal{A}_{j,\alpha}(\tau) + \sum_{k=0}^{n-1}\mathcal{D}'_\varepsilon[n,k+1]\big[\mathcal{A}_{j,\alpha}, \mathcal{D}'_\varepsilon[k+1,k]\big]\mathcal{D}'_\varepsilon[k,0], ,$$

where we set $\mathcal{D}'_\varepsilon[k,k] = \mathrm{id}$. Using Eqs. 19 and 20, we obtain that

$$\mathcal{A}_{j,\alpha}(\tau)\mathcal{D}'_\varepsilon[n,0] = \mathcal{D}'_\varepsilon[n,0]\mathcal{A}_{j,\alpha}(\tau) - i\sum_{k=0}^{n-1}\mathcal{D}'_\varepsilon[n,k+1]\bigg(\int_{k\varepsilon}^{(k+1)\varepsilon} K_{j,\alpha}(\tau - s)L_{j,\alpha}ds\bigg)\mathcal{D}'_\varepsilon[k,0].$$

Taking the limit of $\varepsilon \to 0$ in this equation while setting $n = \lceil t/\varepsilon\rceil$, we obtain the lemma.

An immediate useful consequence of lemma 12 is the following lemma.

*Lemma* 13. Suppose $\mathcal{E}'(t,s) = \mathcal{T}\exp(\int_s^t \mathcal{L}'(\tau)d\tau)$ is the channel generated by the Lindbladian $\mathcal{L}'(t)$ in Eq. 16, and let $\rho(0)$ be an initial state which is vacuum in the decohering environment, then

$$\|\mathrm{Tr}_E(A_{j,\alpha}(t)\mathcal{E}'(t,0)(\rho(0)))\|_{\mathrm{op},1} \leq 2\sqrt{\delta},$$

where $\mathrm{Tr}_E$ is a partial trace over the decohering environment.

*Proof.* This lemma can be proved by using lemma 12. Since $\rho(0)$ is in the vacuum state in the environment, from lemma 12 it follows that

$$A_{j,\alpha}(t)\mathcal{E}'(t,0)(\rho(0)) = -i\int_0^s K_{j,\alpha}(t-t')\mathcal{E}'(t,t')([L_{j,\alpha}, \mathcal{E}'(t',0)(\rho(0))])dt', \tag{21}$$

where we have used that $A_{j,\alpha}(s)\rho(0) = 0$. Using Eqs. 18 and 21 and the fact that quantum channels are contraction with respect to the one norm, we then obtain that

$$\|A_{j,\alpha}(s)\mathcal{E}'(s,0)(\rho(0))\|_{\mathrm{op},1} \leq \sup_{s'\in[0,s]}\|\mathcal{E}'(s,s')(L_{j,\alpha}\mathcal{E}'(s,0)(\rho(0)))\|_{\mathrm{op},1}\, ds' \leq \|L_{j,\alpha}\| \leq \sqrt{\delta}. \tag{22}$$

Noting that $\mathrm{Tr}_E$ is again a contraction with respect to the one norm, we obtain the lemma statement.

*Lemma* 14. For any $t > 0$ and $s \in [0,t]$, initial state $\rho(0)$ which is vacuum in the decohering environment and local operator $O$ supported in $S_O$,

$$\left|\mathrm{Tr}\bigg(Oe^{\mathcal{L}(t-s)}(\mathcal{L}'_\alpha - \mathcal{L}_\alpha)\mathcal{E}'(s,0)(\rho(0))\bigg)\right| \leq \delta|S_O|\|O\|\min\big((e^{vt}-1)e^{-\mu d(S_O,\Lambda_\alpha)}, 1\big), \text{ and}$$

$$\left|\mathrm{Tr}\bigg(Oe^{\mathcal{L}(t-s)}\big([h_{\mathrm{SE},\alpha}(s), \mathcal{E}'(s,0)(\rho(0))]\big)\bigg)\right| \leq 4n_L\delta|S_O|\|O\|\min\big((e^{vt}-1)e^{-\mu d(S_O,\Lambda_\alpha)}, 1\big).$$

*Proof.* The first bound follows directly from the Lieb-Robinson's bound from lemma 11. Note that the super-operator $(\mathcal{L}_\alpha - \mathcal{L}'_\alpha)^\dagger$ has $I$ in its null space, and $\|(\mathcal{L}_\alpha - \mathcal{L}'_\alpha)^\dagger\|_{\infty\to\infty,cb} = \|\mathcal{L}_\alpha - \mathcal{L}'_\alpha\|_\diamond \leq \delta$. Therefore, it follows from that

$$\begin{aligned}\left|\text{Tr}\left(Oe^{\mathcal{L}(t-s)}(\mathcal{L}'_\alpha - \mathcal{L}_\alpha)\mathcal{E}'(s,0)(\rho(0))\right)\right| &= \left|\text{Tr}\left((\mathcal{L}'_\alpha - \mathcal{L}_\alpha)^\dagger(e^{\mathcal{L}^\dagger(t-s)}(O))\mathcal{E}'(s,0)(\rho(0))\right)\right|, \\ &\leq \|(\mathcal{L}'_\alpha - \mathcal{L}_\alpha)^\dagger(e^{\mathcal{L}^\dagger(t-s)}(O))\|, \\ &\leq \delta|S_O|\|O\|\min(e^{-\mu d(S_O,\Lambda_\alpha)}(e^{vt}-1),1),\end{aligned}$$

where we have used lemma 11 in the last step.

Next, we provide a similar upper bound on $\text{Tr}(Oe^{\mathcal{L}(t-s)}([h_{\text{SE},\alpha},\mathcal{E}'(s,0)(\rho(0))]))$. Using the explicit expression for $h_{\text{SE},\alpha}(t)$, we obtain that

$$\begin{aligned}\left|\text{Tr}(Oe^{\mathcal{L}(t-s)}([h_{\text{SE},\alpha},\mathcal{E}'(s,0)(\rho(0))]))\right| &= \left|\sum_{j=1}^{n_L}\left(\text{Tr}_S(Oe^{\mathcal{L}(t-s)}([L^\dagger_{j,\alpha},\text{Tr}_E\big(A_{j,\alpha}(s)\mathcal{E}'(s,0)(\rho(0))]])) - \text{h.c.}\right)\right|, \\ &\leq 2\sum_{j=1}^{n_L}\left|\text{Tr}_S\big(Oe^{\mathcal{L}(t-s)}([L^\dagger_{j,\alpha},\text{Tr}_E\big(A_{j,\alpha}(s)\mathcal{E}'(s,0)(\rho(0))]]))\right|, \\ &= 2\sum_{j=1}^{n_L}\left|\text{Tr}_S\big([e^{\mathcal{L}^\dagger(t-s)}(O),L^\dagger_{j,\alpha}]\text{Tr}_E\big(A_{j,\alpha}(s)\mathcal{E}'(s,0)(\rho(0))\big)\big)\right|, \\ &\leq 2\sum_{j=1}^{n_L}\|[e^{\mathcal{L}^\dagger(t-s)}(O),L^\dagger_{j,\alpha}]\|\,\big\|\text{Tr}_E\big(A_{j,\alpha}(s)\mathcal{E}'(s,0)(\rho(0))\big\|_1\end{aligned}\tag{23}$$

where $\text{Tr}_E$ is the partial trace over the decohering environment, and by $\text{Tr}_S$ is the partial trace over the system. Now, if the initial state $\rho(0)$ is vacuum in the environment then from lemma 12 we obtain

$$|\text{Tr}_S(Oe^{\mathcal{L}(t-s)}([L^\dagger_{j,\alpha},\text{Tr}_E\big(A_{j,\alpha}(s)\mathcal{E}'(s,0)(\rho(0))]]))| \leq \left\|[e^{\mathcal{L}^\dagger(t-s)}(O),L^\dagger_{j,\alpha}]\right\|\,\big\|\text{Tr}_E\big(A_{j,\alpha}(s)\mathcal{E}'(s,0)(\rho(0))\big\|_{\text{op},1}. \tag{24}$$

We note that since the super operator $[\cdot,L^\dagger_{j,\alpha}]$ is supported only on $\Lambda_\alpha$ and satisfies $[I,L^\dagger_{j,\alpha}] = 0$ together with $\|[\,\cdot\,,L^\dagger_{j,\alpha}]\|_{\infty\to\infty,cb} \leq 2\|L_{j,\alpha}\| \leq 2\sqrt{\delta}$. Therefore, from the Lieb-Robinson bound in lemma 11, we obtain the bound

$$\|[O,L^\dagger_{j,\alpha}]\| \leq 2\sqrt{\delta}|S_O|\|O\|\min\big(e^{-\mu d(S_O,\Lambda_\alpha)}(e^{vt}-1),1\big). \tag{25}$$

From Eqs. 23, 24, 25 and lemma 13, we then obtain

$$\left|\text{Tr}(Oe^{\mathcal{L}(t-s)}([h_{\text{SE},\alpha}(s),\mathcal{E}'(s,0)(\rho(0))])])\right| \leq 4n_L\delta|S_O|\|O\|\min\big(e^{-\mu d(S_O,\Lambda_\alpha)}(e^{vt}-1),1\big).$$

*Proof (of proposition 5).* Suppose $\mathcal{O} = \text{Tr}[Oe^{\mathcal{L}t}(\rho(0))]$ and $\mathcal{O}' = \text{Tr}[O\mathcal{E}'(t,0)(\rho(0))]$ are the observable expectation values in the noiseless and noisy settings respectively. We can express the error in the observable as

$$\begin{aligned}|\mathcal{O}'-\mathcal{O}| = |\text{Tr}[O(\mathcal{E}'(t,0)(\rho(0)) - e^{\mathcal{L}t}(\rho(0)))]| &\leq \int_0^t\left|\text{Tr}\left(Oe^{\mathcal{L}(t-s)}(\mathcal{L}'(s)-\mathcal{L})\mathcal{E}'(s,0)(\rho(0))\right)\right|ds, \\ \leq \sum_\alpha\bigg(\int_0^t\left|\text{Tr}\left(Oe^{\mathcal{L}(t-s)}(\mathcal{L}'_\alpha-\mathcal{L}_\alpha)\mathcal{E}'(s,0)(\rho(0))\right)\right|ds &+ \int_0^t\left|\text{Tr}\left(Oe^{\mathcal{L}(t-s)}[h_{\text{SE},\alpha}(s),\mathcal{E}'(s,0)(\rho(0))]\right)\right|ds\bigg).\end{aligned}$$

Using lemma 14, we obtain that

$$|\mathcal{O}-\mathcal{O}'| \leq \delta t|S_O|\|O\|(1+4n_L)\sum_\alpha\max\big(e^{-\mu_\alpha d(S_O,\Lambda_\alpha)}(e^{vt}-1),1\big) \leq O(\delta t^{d+1}).$$

### B. Proof of proposition 6 (Ground states of gapped local Hamiltonians)

We will apply the formalism developed in Ref. [4] for spectral flows for families of gapped Hamiltonians. We are interested in a target spatially local Hamiltonian $H$, expressed as

$$H = \sum_{x \in \mathfrak{L}} h_x,$$

where $h_x$ acts only on spins with a distance $R$ of $x \in \mathfrak{L}$, and $\|h_x\| \leq 1$. The implemented Hamiltonian $H'$ is assumed to have a similar form,

$$H' = \sum_{x \in \mathfrak{L}} \big(h_x + v_x\big),$$

where $\|v_x\| \leq \delta$ for all $x \in \mathfrak{L}$. We assume that $H$ is stably gapped with gap $\Delta$ i.e. any $H'$ of the above form has an energy gap between the ground state and the first excited state that is larger than $\Delta$. We consider the family of Hamiltonians, $H_s$, for $s \in [0, 1]$, defined by

$$H_s = H + s(H' - H) = \sum_{x \in \mathfrak{L}} h_x + s v_x,$$

and note that the assumption of being stably gapped is equivalent to $H_s$ being gapped, with the gap being larger than $\Delta$, for all $s \in [0, 1]$. Now, the spectral flow method allows us to construct a unitary $U(s)$ that relates the ground state $|G_{s=0}\rangle$ of $H_{s=0} = H$ to the ground state $|G_s\rangle$ of $H_s$ as provided in the following lemma.

*Lemma* 15 (From Ref. [4]). Consider the unitary $U(s)$ obtained from

$$\frac{d}{ds} U(s) = i D(s) U(s) \text{ where } D(s) = \int_{-\infty}^{\infty} W_\Delta(t) e^{-itH_s} (H' - H) e^{itH_s} dt$$

where $W_\Delta \in L^1(\mathbb{R})$ is a real valued odd function which satisfies

(a) $|W_\Delta(t)|$ is bounded and satisfies

$$\|W_\Delta\|_\infty = \sup_{t \in \mathbb{R}} |W_\Delta(t)| = \frac{1}{2}. \tag{26}$$

(b) For $t > 0$, the function $I_\Delta(t) = \int_t^\infty |W_\Delta(s)| ds$ satisfies

$$I_\Delta(t) \leq G(\Delta t), \tag{27}$$

where $G(x)$ falls off faster than any polynomial as $x \to \infty$.

Then, $|G_s\rangle = U(s) |G_{s=0}\rangle$, where $|G_s\rangle$ is the ground state of $H(s)$.

*Proof (of proposition 6).* Using this result, we can straightforwardly show the stability of the quantum simulation task of computing a local observable in the ground state of $H$. To see this, we note that

$$|\langle G_0| O |G_0\rangle - \langle G_s| O |G_s\rangle| = \left| \langle G_0| \Big(O - U^\dagger(s) O U(s)\Big) |G_0\rangle \right| \leq \|O - U^\dagger(s) O U(s)\| \leq \int_0^s \|[O, D(s')]\| ds'.$$

It then remains to bound $\|[O, D(s')]\|$ — we can do this by following lemma 4.7 in Ref. [4], and we reproduce this below — we start by noting that

$$\|[O, D(s')]\| \leq \sum_{x \in \mathfrak{L}} \left\| \int_{-\infty}^{\infty} W_\Delta(t) [O, e^{itH_s} v_x e^{-itH_s}] dt \right\|.$$

For each term in this summation, we further split the integral and bound it as

$$\left\| \int_{-\infty}^{\infty} W_\Delta(t) [O, e^{itH_s} v_x e^{-itH_s}] dt \right\| \leq \left\| \int_{|t| \leq T_x} W_\Delta(t) [O, e^{itH_s} v_x e^{-itH_s}] dt \right\| + \left\| \int_{|t| > T_x} W_\Delta(t) [O, e^{itH_s} v_x e^{-itH_s}] dt \right\|.$$

For the first term, which only concerns with $|t| \leq T_x$, we use the Lieb-Robinson bound (lemma 11) and Eq. 26 to obtain

$$\left\| \int_{|t|\leq T_x} W_\Delta(t)[O, e^{itH_s} v_x e^{-itH_s}]dt \right\| \leq \|O\|\|v_x\||S_O|e^{-\mu d(S_O, S_{v_x})} \int_0^{T_x} (e^{vt} - 1)dt \leq \|O\|\|v_x\||S_O| \frac{e^{-\mu d(S_O, S_{v_x})} e^{vT_x}}{v}.$$

For the second term for $|t| \geq T_x$, we use Eq. 27 together with the fact that $W_\Delta$ is an odd function and the simple bound $\|[O, e^{itH_s} v_x e^{-itH_s}]\| \leq 2\|O\|\|v_x\|$ to obtain that

$$\left\| \int_{|t|\geq T_x} W_\Delta(t)[O, e^{itH_s} v_x e^{-itH_s}]dt \right\| \leq 2\|O\|\|v_x\| \int_{|t|\geq T_x} |W_\Delta(t)| \leq 4\|O\|\|v_x\|G(\Delta T_x),$$

Note that $T_x$ can be arbitrary in the above two estimates — choosing $T_x = \mu d(S_O, S_{v_x})/2v$, we obtain that

$$\left\| \int_{-\infty}^{\infty} W_\Delta(t)[O, e^{itH_s} v_x, e^{-itH_s}]dt \right\| \leq \|O\|\|v_x\| \left[ \frac{|S_O|}{v} e^{-\mu d(S_O, S_{v_x})/2} + 2G\left( \frac{\Delta\mu}{v} d(S_O, S_{v_x}) \right) \right],$$

and therefore, for all $s' \in [0, s]$, we obtain the bound

$$\|[O, D(s')]\| \leq \|O\|\delta \sum_{x\in\mathfrak{L}} \left[ \frac{|S_O|}{v} e^{-\mu d(S_O, S_{v_x})/2} + 2G\left( \frac{\Delta\mu}{v} d(S_O, S_{v_x}) \right) \right].$$

Noting that the summand in the above expression decreases faster than any polynomial in $d(S_O, S_{v_x})$, we see that it will be upper bounded by a constant independent of the size of the lattice $\mathfrak{L}$, thus independent of $n$. This proves the proposition. □

### C. Proof of proposition 7 (Gibbs state with exponential clustering of correlations)

We begin by presenting a proof of a standard bound on the perturbation of Gibbs states of a Hamiltonian. This follows from [5], and we state it here for the convenience of the reader.

*Lemma* 16. [Follows from Ref. [5]] Given bounded Hermitian operators $H$ and $V$, and any bounded Hermitian operator (observable) O,

$$\left| \text{Tr}\left( \frac{Oe^{-\beta H}}{Z_H(\beta)} \right) - \text{Tr}\left( \frac{Oe^{-\beta(H+V)}}{Z_{H+V}(\beta)} \right) \right| \leq 2\|O\| \sqrt{1 - \exp(-\beta\|V\|)},$$

*Proof.* This is a straightforward application of the result in appendix C of Ref. [5], in which they show that

$$\mathcal{F}\left( \frac{e^{-\beta H}}{\text{Tr}(e^{-\beta H})}, \frac{e^{-\beta H'}}{\text{Tr}(e^{-\beta H'})} \right) \leq \exp(-\beta\|V\|),$$

where $F(\rho_1, \rho_2) = \|\sqrt{\rho_1}\sqrt{\rho_2}\|_{\text{op},1}$ is the fidelity between $\rho_1$ and $\rho_2$. Note also that $\|\rho_1 - \rho_2\|_{\text{op, 1}} \leq 2\sqrt{1 - F(\rho_1, \rho_2)}$, and therefore

$$\left| \text{Tr}\left( \frac{Oe^{-\beta H}}{Z_H(\beta)} \right) - \text{Tr}\left( \frac{Oe^{-\beta(H+V)}}{Z_{H+V}(\beta)} \right) \right| \leq \|O\| \left\| \frac{e^{-\beta H}}{\text{Tr}(e^{-\beta H})} - \frac{e^{-\beta H'}}{\text{Tr}(e^{-\beta H'})} \right\|_{\text{op},1} \leq 2\sqrt{1 - e^{-\beta\|V\|}}$$

We next need the notion of exponentially-clustered correlations in a Gibbs state — which we reproduce below from Ref. [6]. We will consider Hamiltonians on $\mathfrak{L} \subset \mathbb{Z}^d$ expressed as

$$H = \sum_{x\in\mathfrak{L}} h_x,$$

where $h_x$ acts only on spins within a distance $R$ of $x \in \mathfrak{L}$ and $\|h_x\| \leq 1$. We will denote by $\text{supp}(h_x) \subseteq \mathfrak{L}$ the support of $h_x$. Given $X \subseteq \mathfrak{L}$, we denote by $H_X$ the operator

$$H_X = \sum_{x|\text{supp}(h_x)\subseteq X} h_x,$$

i.e. $H_X$ is the Hamiltonian $H$ obtained on restricting $H$ to the set $X$.

*Definition* 1. A local Hamiltonian $H$ is said to have exponential clustering of correlations at inverse temperature $\beta$ if $\exists c_1, c_2 > 0$ such that for all $X \subset \mathfrak{L}$ and operators $A, B$ with $\text{supp}(A), \text{supp}(B) \subset X$ with $d(\text{supp}(A), \text{supp}(B)) \geq l$,

$$\left|\text{Tr}\big((A \otimes B)\sigma_X(\beta)\big) - \text{Tr}\big(A\sigma_X(\beta)\big)\text{Tr}\big(B\sigma_X(\beta)\big)\right| \leq c_2\|A\|\|B\|e^{-c_1 l},$$

where $\sigma_X(\beta) = e^{-\beta H_X}/\text{Tr}[e^{-\beta H_X}]$ is the Gibbs state corresponding to $H_X$ at inverse temperature $\beta$.

An important property of Hamiltonians with exponential clustering of correlations, which relies on quantum belief propagation [7] and is proved in Ref. [6], is that local observables can be estimated locally.

*Lemma* 17 (From Ref. [6]). Suppose $H$ is a local Hamiltonian on a finite lattice $\mathfrak{L} \subset \mathbb{Z}^d$ with exponential clustering of correlations at inverse temperature $\beta$. If $\mathfrak{L} = \mathcal{A} \cup \mathcal{B} \cup \mathcal{C}$ such that $\text{dist}(\mathcal{A}, \mathcal{C}) \geq l$, then $\exists c_1', c_2'$ such that

$$\|\text{Tr}_{\mathcal{B},\mathcal{C}}(\sigma_{\mathfrak{L}}(\beta)) - \text{Tr}_{\mathcal{B}}(\sigma_{\mathcal{A}\cup\mathcal{B}}(\beta))\|_{\text{tr}} \leq |\partial\mathcal{C}|c_2' e^{-c_1' l},$$

where $\sigma_X(\beta)$ is the Gibbs state corresponding to $H_X$ and $\partial\mathcal{C}$ is the boundary between $B, C$.

*Proof (of proposition 7).* We assume that both $H$ and $H'$ have exponential clustering of correlations and satisfy lemma 17. Suppose $O$ is a local observable with support $S_O$ and consider $\mathcal{B}$ to be a region around $S_O$ and $\mathcal{C}$ be the remainder of the lattice. We also assume that $d(\mathcal{C}, S_O) \geq l$, for some $l$ to be chosen later. We denote by $\sigma_l(\beta)$ and $\sigma_l'(\beta)$ the Gibbs state, at inverse temperature $\beta$, corresponding to $H_{S_O\cup\mathcal{B}}$ and $H'_{S_O\cup\mathcal{B}}$ respectively, and by $\sigma(\beta), \sigma'(\beta)$ the Gibbs state corresponding to $H$ and $H'$. Now, from lemma 17 it follows that

$$|\text{Tr}(O\sigma(\beta)) - \text{Tr}(O\sigma_l(\beta))|, |\text{Tr}(O\sigma'(\beta)) - \text{Tr}(O\sigma_l'(\beta))| \leq \|O\|d(2l + R_O)^{d-1}c_2' e^{-c_1' l},$$

where $R_O = \text{diam}(S_O)$ and we have used that $|\partial\mathcal{C}| \leq d \times \text{diam}(S_O \cup \mathcal{B})^{d-1} \leq d(2l + R_O)^{d-1}$. Furthermore, lemma 16 can be used to bound $|\text{Tr}(O\sigma_l(\beta)) - \text{Tr}(O\sigma_l'(\beta))|$. We note that $\|H_{S_O\cup\mathcal{B}} - H'_{S_O\cup\mathcal{B}}\| \leq \delta(2l + R_O)^d$ Therefore,

$$|\text{Tr}(O\sigma_l(\beta)) - \text{Tr}(O\sigma_l'(\beta))| \leq 2\|O\|\sqrt{1 - \exp(-\beta\|H_{S_O\cup\mathcal{B}} - H'_{S_O\cup\mathcal{B}}\|)} \leq 2\|O\|\sqrt{1 - \exp(-\beta(2l + R_O)^d\delta)}.$$

Thus, from the triangle inequality we obtain the bound that

$$|\text{Tr}(O\sigma(\beta)) - \text{Tr}(O\sigma'(\beta))| \leq \|O\|\left[d(2l + R_O)^{d-1}c_2' e^{-c_1' l} + 2\sqrt{1 - \exp(-\beta(2l + R_O)^d\delta)}\right].$$

For $\beta = \Theta(1)$, choosing $2l + R_O = {c_1'}^{-1}\log(1/\delta)$, we obtain that

$$|\text{Tr}(O\sigma(\beta)) - \text{Tr}(O\sigma'(\beta))| \leq \|O\|O\big(\delta \log^d(1/\delta)\big),$$

which proves the proposition. □

### D. Fixed point of rapidly mixing Lindbladians

In this section, we consider spatially local Lindbladians which are also rapidly mixing as defined in Ref. [8]. We establish that local observables measured in the fixed point are robust to both coherent and incoherent errors. We point out that Ref. [8] already proved this statement if the incoherent errors are considered to be Markovian — we show that this stability of local observables is retained in the more general setting of incoherent non-Markovian errors. We first reproduce the definition of rapidly mixing Lindbladians from Ref. [8].

*Assumption* 1. Suppose $\mathcal{L} = \sum_\alpha \mathcal{L}_\alpha$ is a spatially local Lindbladian on $\mathbb{Z}_L^d$ where $\mathcal{L}_\alpha$ are supported in $S_\alpha$, and let $\mathcal{L}_{\Lambda_l} = \sum_{\alpha: S_\alpha \subseteq \Lambda_l} \mathcal{L}_\alpha$ be the Lindbladian obtained from $\mathcal{L}$ by only retaining $\mathcal{L}_\alpha$ which are supported entirely in sublattice $\Lambda_l \cong \mathbb{Z}_l^d$. Then, for any choice of $\Lambda_l$, $\mathcal{L}_{\Lambda_l}$ has a unique fixed point $\sigma_{\Lambda_l}$ and

$$\|e^{\mathcal{L}_{\Lambda_l} t}(\cdot) - \sigma_{\Lambda_l}\text{Tr}(\cdot)\|_\diamond \leq \kappa(l)e^{-\gamma t},$$

where $\gamma > 0$ is some constant and $\kappa(l) \leq O(\text{poly}(l))$.

An important consequence of this assumption, established in Ref. [8], is the local rapid mixing property which we state below.

*Lemma* 18 (Ref. [8]). Suppose $\mathcal{L} = \sum_\alpha \mathcal{L}_\alpha$ is a spatially local Lindbladian which satisfies assumption 1 and $O$ is a local observable with support $S_O$. Then,

$$\|e^{\mathcal{L}^\dagger t}(O) - I\ \text{Tr}(\sigma O)\| \leq \|O\| k(|S_O|) e^{-\gamma t},$$

for some $k(l) \leq O(\text{poly}(l))$.

*Proof (of proposition 8)* We will show that for any time $t > 0$, the observable obtained by the noisy quantum simulator is close to the true observable if assumption 1 is satisfied. Denoting by $\mathcal{O}$ the true observable, and by $\mathcal{O}'$ the observable in the presence of errors and noise, we start with

$$\begin{aligned}
|\mathcal{O} - \mathcal{O}'| &= \left|\int_0^t \text{Tr}\Big(e^{\mathcal{L}^\dagger(t-s)}(O)(\mathcal{L}'(s) - \mathcal{L})(\mathcal{E}'(s,0)(\rho(0)))\Big) ds\right|, \\
&\leq \sum_\alpha \int_0^t \left|\text{Tr}\Big(e^{\mathcal{L}^\dagger(t-s)}(O)\mathcal{L}_\alpha(s)(\mathcal{E}'(s,0)(\rho(0)))\Big)\right| ds, \\
&\leq \sum_\alpha \int_0^t \left|\text{Tr}\Big(e^{\mathcal{L}^\dagger s}(O)\mathcal{L}_\alpha(t-s)(\mathcal{E}'(t-s,0)(\rho(0)))\Big)\right| ds, \\
&\leq \sum_\alpha \big(e_{\alpha,<}(t_\alpha, t) + e_{\alpha,>}(t_\alpha, t)\big). 
\end{aligned} \tag{28a}$$

where $t_\alpha$ remains to be chosen and

$$e_{\alpha,<}(t_\alpha, t) = \left|\int_0^{\min(t,t_\alpha)} \text{Tr}\Big(e^{\mathcal{L}^\dagger s}(O)\mathcal{L}'_\alpha(t-s)(\mathcal{E}'(t-s,0)(\rho(0)))\Big) ds\right| \text{ and} \tag{28b}$$

$$e_{\alpha,>}(t_\alpha, t) = \left|\int_{\min(t,t_\alpha)}^t \text{Tr}\Big(e^{\mathcal{L}^\dagger s}(O)\mathcal{L}'_\alpha(t-s)(\mathcal{E}'(t-s,0)(\rho(0)))\Big) ds\right| \tag{28c}$$

We now separately upper bound both $e_<(t_\alpha, t)$ and $e_>(t_\alpha, t)$. We can use lemma 14 to obtain

$$e_{\alpha,<}(t_\alpha, t) \leq \delta t_\alpha |S_O| \|O\| (1 + 4n_L) \min(e^{-\mu d(S_O, \Lambda_\alpha)}(e^{v t_\alpha} - 1), 1). \tag{29}$$

Next, consider $e_{\alpha,>}(t_\alpha, t)$ — for $t_\alpha < t$, it trivially follows that $e_{\alpha,>}(t_\alpha, t) = 0$. For $t_\alpha \geq t$, we use lemma 18 — we begin by expressing $e^{\mathcal{L}^\dagger s}(O) = \text{Tr}(\sigma O)I + (e^{\mathcal{L}^\dagger s}(O) - \text{Tr}(\sigma O)I)$. From this decomposition and the definition of $e_{\alpha,>}(t_\alpha, t)$, we obtain that

$$\begin{aligned}
e_{\alpha,>}(t_\alpha, t) = \Bigg| &\int_{\min(t,t_\alpha)}^t \text{Tr}(O\sigma)\text{Tr}\Big(\mathcal{L}'_\alpha(t-s)\big(\mathcal{E}'(t-s,0)(\rho(0))\big)\Big) ds + \\
&\int_{\min(t,t_\alpha)}^t \text{Tr}\Big(\big(e^{\mathcal{L}^\dagger s}(O) - \text{Tr}(O\sigma)I\big)\mathcal{L}'_\alpha(t-s)\big(\mathcal{E}'(t-s,0)(\rho(0))\big)\Big) ds\Bigg|, \\
= &\left|\int_{\min(t,t_\alpha)}^t \text{Tr}\Big(\big(e^{\mathcal{L}^\dagger s}(O) - \text{Tr}(O\sigma)I\big)\mathcal{L}'_\alpha(t-s)\big(\mathcal{E}'(t-s,0)(\rho(0))\big)\Big) ds\right|, 
\end{aligned} \tag{30}$$

where we have used the fact that since $\mathcal{L}'_\alpha(t-s)$ is a valid Lindbladian, it maps any operator to a traceless operator — in particular, $\text{Tr}(\mathcal{L}'_\alpha(t-s)(\mathcal{E}'(t-s,0)(\rho(0)))) = 0$. Next, we note that since $O$ is a system operator and $\mathcal{L}$ (the target Lindbladian) is also only defined on the system, $e^{\mathcal{L}^\dagger s}(O) - \text{Tr}(O\sigma)I$ is an operator that does not act on the environment. Therefore, we obtain from Eq. 30 that

$$\begin{aligned}
e_{\alpha,>}(t_\alpha, t) &= \left|\int_{\min(t,t_\alpha)}^t \text{Tr}_S\Big(\big(e^{\mathcal{L}^\dagger s}(O) - \text{Tr}(O\sigma)I\big)\text{Tr}_E\Big(\mathcal{L}'_\alpha(t-s)\big(\mathcal{E}'(t-s,0)(\rho(0))\big)\Big)\Big) ds\right|, \\
&\leq \int_{\min(t,t_\alpha)}^t \|e^{\mathcal{L}^\dagger s}(O) - \text{Tr}(O\sigma)I\| \|\text{Tr}_E(\mathcal{L}'_\alpha(t-s)(\mathcal{E}'(t-s,0)(\rho(0)))\|_{\text{op},1} ds, \\
&\leq \|O\| k(|S_O|) \int_{\min(t,t_\alpha)}^t e^{-\gamma s} \|\text{Tr}_E(\mathcal{L}'_\alpha(t-s)(\mathcal{E}'(t-s,0)(\rho(0)))\|_{\text{op},1} ds,
\end{aligned}$$

where, in the last step, we have used lemma 18. Now, using the explicit expression for $\mathcal{L}'_\alpha(s)$, we obtain that

$$e_{\alpha,>}(t_\alpha, t) \leq \|O\|k(|S_O|)\bigg(\int_{\min(t,t_\alpha)}^{t} e^{-\gamma s}\,\|\text{Tr}_E((\mathcal{L}_\alpha - \mathcal{L}'_\alpha)(\mathcal{E}'(t-s,0)(\rho(0)))\|_{\text{op},1}\,ds + \\ 2\sum_{j=1}^{n_L}\int_{\min(t,t_\alpha)}^{t} e^{-\gamma s}\|L_{j,\alpha}^\dagger \text{Tr}_E(A_{j,\alpha}\mathcal{E}'(t-s,0)(\rho(0)))\|_{\text{op},1}\bigg).$$

Using $\|\mathcal{L}_\alpha - \mathcal{L}'_\alpha\|_\diamond \leq \delta$, $\|L_{j,\alpha}\| \leq \sqrt{\delta}$ and lemma 13, we obtain that

$$e_{\alpha,>}(t_\alpha, t) \leq \|O\|k(|S_O|)\delta\big(1+2n_L\big)\int_{\min(t,t_\alpha)}^{t} e^{-\gamma s}ds \leq \|O\|k(|S_O|)\delta\big(1+2n_L\big)e^{-\gamma t_\alpha}. \tag{31}$$

Finally, from Eqs. 28a, 29 and 31, we obtain that

$$|\mathcal{O} - \mathcal{O}'| \leq \|O\|\delta(1+4n_L)\sum_\alpha \bigg(k(|S_O|)e^{-\gamma t_\alpha} + |S_O|t_\alpha \min\big(e^{-\mu d(S_O,\Lambda_\alpha)}\big(e^{vt_\alpha}-1\big),1\big)\bigg).$$

Now, we make a choice of $t_\alpha = \mu d(S_O, \Lambda_\alpha)/2v$, we obtain that

$$|\mathcal{O} - \mathcal{O}'| \leq \|O\|\delta(1+4tn_L)\sum_\alpha \bigg(k(|S_O|)e^{-\gamma\mu d(S_O,\Lambda_\alpha)/2v} + |S_O|\frac{\mu d(S_O,\Lambda_\alpha)}{2v}e^{-\mu d(S_O,\Lambda_\alpha)/2}\bigg).$$

The summation in the above bound converges to a constant independent of the system size, and we thus obtain that $|\mathcal{O} - \mathcal{O}'| \leq O(\delta)$. □

.

## Supplemental note IV: Lower bounds on convergence to thermodynamic limits

### A. Dynamics

In the main text, we had considered a sequence of spatially local, translationally invariant Lindbladians $\{\mathcal{L}_n\}$, where $\mathcal{L}_n$ acts on $n$ qudits arranged on the $d-$dimensional lattice $\mathbb{Z}_L^d$ with $n = L^d$. From the Lieb-Robinson bounds, it followed that for local observables $O_n$ at a fixed ($n-$independent) site on the lattice, $\lim_{n\to\infty}\text{Tr}(O_n e^{\mathcal{L}_n t}((|0\rangle\langle 0|)^{\otimes n}))$ exists and, for $t \leq O(1)$, and converges exponentially in $n$ i.e.

$$\Big|\text{Tr}(O_n e^{\mathcal{L}_n t}((|0\rangle\langle 0|)^{\otimes n})) - \lim_{n\to\infty}\text{Tr}(O_n e^{\mathcal{L}_n t}((|0\rangle\langle 0|)^{\otimes n}))\Big| \leq O(e^{-\Omega(n^{1/d})}). \tag{32}$$

In particular, this convergence estimate implies that $\big|\text{Tr}(O_n e^{\mathcal{L}_n t}((|0\rangle\langle 0|)^{\otimes n})) - \lim_{n\to\infty}\text{Tr}(O_n e^{\mathcal{L}_n t}((|0\rangle\langle 0|)^{\otimes n}))\big| \leq \varepsilon$ if $n \geq \Omega(\log^d(\varepsilon^{-1}))$. In this subsection, we will exhibit a simple model on a $d-$dimensional lattice almost saturates this bound. We first establish some useful technical lemmas.

*Lemma* 19. For $p \in \mathbb{N}$, it follows that

(a) If $L \in \mathbb{N}$ is even then,

$$\sum_{m=0}^{L-1}\sin^{2p}\left(\frac{m\pi}{L}\right) = \frac{L}{2^{2p}}\binom{2p}{p} + \frac{L}{2^{2p-1}}\sum_{1\leq k\leq p/L}\binom{2p}{p+kL}.$$

(b) The integral

$$\int_0^\pi \sin^{2p}(\theta)d\theta = \frac{\pi}{2^{2p}}\binom{2p}{p}.$$

*Proof*: (a) We begin by noting that

$$\sum_{m=0}^{L-1}\sin^{2p}\left(\frac{\pi m}{L}\right) = 2\sum_{m=0}^{L/2-1}\cos^{2p}\left(\frac{m\pi}{L}\right) - 1. \tag{33}$$

Next, we have that

$$\sum_{m=0}^{L/2-1} \cos^{2p}\left(\frac{\pi m}{L}\right) = \frac{1}{2^{2p}} \sum_{m=0}^{L/2-1} \sum_{k=0}^{2p} \binom{2p}{k} \mathrm{Re}\Big(e^{i2\pi m(p-k)/L}\Big) = \frac{1}{2^{2p}} \sum_{k=0}^{2p} \binom{2p}{k} \sum_{m=0}^{L/2-1} \mathrm{Re}\Big(e^{i2\pi m(p-k)/L}\Big).$$

Noting that

$$\sum_{n=0}^{L/2-1} \mathrm{Re}\Big(e^{i2\pi nk/L}\Big) = \begin{cases} N/2 & \text{if } k \in \{0, \pm L, \pm 2L \dots\}, \\ 0 & \text{if } k \text{ is odd}, \\ 1 & \text{if } k \text{ is even and } k \notin \{0, \pm L, \pm 2L \dots\}, \end{cases}$$

we obtain

$$\sum_{n=0}^{L/2-1} \cos^{2p}\left(\frac{\pi n}{L}\right) = \frac{L}{2^{2p+1}} \binom{2p}{p} + \frac{L}{2^{2p}} \sum_{1 \le k \le p/L} \binom{2p}{p+k} + \frac{1}{2}. \tag{34}$$

Part (a) of the lemma then follows from Eqs. 33 and 34.

(b) We note that

$$\int_0^{\pi} \sin^{2p}(\theta) d\theta = 2 \int_0^{\pi/2} \cos^{2p}(\theta) d\theta = \frac{2}{2^{2p}} \sum_{m=0}^{2p} \binom{2p}{m} \int_0^{\pi/2} \mathrm{Re}\Big(e^{i2(p-m)\theta}\Big) d\theta.$$

Noting that for $q \in \mathbb{Z}$,

$$\int_0^{\pi/2} \mathrm{Re}\big(e^{2iq\theta}\big) d\theta = \begin{cases} \pi/2 & \text{if } q = 0, \\ 0 & \text{otherwise}, \end{cases}$$

and thus

$$\int_0^{\pi} \sin^{2p}(\theta) d\theta = \frac{\pi}{2^{2p}} \binom{2p}{p}.$$

□

*Lemma* 20. For $0 \le \alpha \le \pi/4$,

$$\frac{1}{L} \sum_{m=-L}^{L-1} \cos\left(\alpha \sin\left(\frac{\pi m}{L}\right)\right) \ge \sqrt{2}.$$

*Proof*: This follows from the simple observation that since $|\sin(\pi m/L)| \le 1$, and the cosine function is decreasing in the interval $[-\pi/2, \pi/2]$, for $0 \le \alpha \le \pi/4$, $\cos(\alpha \sin(\pi m/n)) \ge 1/\sqrt{2}$ for all $m \in \{-L, -L+1 \dots L-1\}$. □

*Lemma* 21. If $L \in \mathbb{N}$ is even and for $0 \le \alpha < 2\sqrt{L}$,

$$\frac{\pi}{L} \sum_{m=-L}^{L-1} \cos\left(\alpha \sin\left(\frac{\pi m}{L}\right)\right) - \int_{-\pi}^{\pi} \cos(\alpha \sin\theta) d\theta \ge 4\pi \frac{(\alpha/2)^{2L}}{(2L)!} \left(1 - \frac{\alpha^2}{4(2L+1)}\right)$$

*Proof*: We begin by using the Taylor expansion of the cosine function to obtain that

$$\frac{\pi}{L} \sum_{m=-L}^{L-1} \cos\left(\alpha \sin\left(\frac{\pi m}{L}\right)\right) - \int_{-\pi}^{\pi} \cos(\alpha \sin\theta) d\theta = \sum_{p=1}^{\infty} (-1)^p \frac{\alpha^{2p}}{(2p)!} \bigg(\frac{\pi}{L} \sum_{m=-L}^{L-1} \sin^{2p}\left(\frac{\pi m}{L}\right) - \int_{-\pi}^{\pi} \sin^{2p}(\theta) d\theta\bigg).$$

Using lemma 19, we then obtain that

$$\frac{\pi}{L} \sum_{m=-L}^{L-1} \cos\left(\alpha \sin\left(\frac{\pi m}{L}\right)\right) - \int_{-\pi}^{\pi} \cos(\alpha \sin\theta) d\theta = \sum_{p=1}^{\infty} T_p(\alpha),$$

where

$$T_p(\alpha) = 4\pi(-1)^p \frac{(\alpha/2)^{2p}}{(2p)!} \sum_{1\leq k\leq p/L} \binom{2p}{p+kL}.$$

Furthermore, assuming that $L$ is even, we note that for even $p$,

$$\begin{aligned} T_p(\alpha) + T_{p+1}(\alpha) &= 4\pi \frac{(\alpha/2)^{2p}}{(2p)!} \sum_{1\leq k\leq p/L} \binom{2p}{p+kL} - 4\pi \frac{(\alpha/2)^{2p+2}}{(2p+2)!} \sum_{1\leq k\leq (p+1)/L} \binom{2p+2}{p+1+kL}, \\ &= 4\pi \frac{(\alpha/2)^{2p}}{(2p)!} \bigg[ \sum_{1\leq k\leq p/L} \binom{2p}{p+kL} - \frac{(\alpha/2)^2}{(2p+2)(2p+1)} \binom{2p+2}{p+1+kL} \bigg], \\ &= 4\pi \frac{(\alpha/2)^{2p}}{(2p)!} \bigg[ \sum_{1\leq k\leq p/L} \binom{2p}{p+kL} \bigg(1 - \frac{\alpha^2}{4((p+1)^2 - k^2L^2)}\bigg) \bigg] \end{aligned}$$

Noting that if $1 \leq k \leq p/L$, $(p+1)^2 - k^2L^2 \geq L$, and for $\alpha \leq 2\sqrt{L}$, $1 - \alpha^2/4((p+1)^2 - k^2L^2) \geq 0$, and we conclude that $T_p(\alpha) + T_{p+1}(\alpha) \geq 0$ for all even $p$.

Next, we note that $T_p(\alpha) = 0$ for $1 \leq p < L$. Since $L$ is even and, as established above, $T_p(\alpha) + T_{p+1}(\alpha) \geq 0$ for even $p$, we obtain the lower bound

$$\frac{\pi}{n} \sum_{m=-L}^{L-1} \cos\left(\alpha \sin\left(\frac{\pi m}{L}\right)\right) - \int_{-\pi}^{\pi} \cos(\alpha \sin\theta) d\theta \geq T_L(\alpha) + T_{L+1}(\alpha) = 4\pi \frac{(\alpha/2)^{2L}}{(2L)!} \left(1 - \frac{\alpha^2}{4(2L+1)}\right) \geq 2\pi \frac{(\alpha/2)^{2L}}{(2L)!},$$

which establishes the lemma. □

The model that we consider is simple fermionic tight-binding model on a $d-$dimensional lattice $\mathfrak{L}^d$, where $\mathfrak{L} = \{-L, -L+1 \dots L-1\}$, with imaginary hopping amplitudes

$$H_n = i \sum_{x\in\mathfrak{L}^d} \sum_{m=1}^{d} \left(a_x^\dagger a_{x+e_m} - \text{h.c.}\right),$$

where $n = (2L)^d$, $e_m$ is the unit vector along the $m^{\text{th}}$ lattice direction and a periodic boundary condition is assumed. For simplicity, we will assume that $L$ is even. This Hamiltonian can be analytically diagonalized to obtain

$$H_n = \sum_{k\in\mathfrak{L}^d} \omega_k \tilde{a}_k^\dagger \tilde{a}_k,$$

where

$$\tilde{a}_k = \frac{1}{(2L)^{d/2}} \sum_{x\in\mathfrak{L}^d} a_x e^{i\pi k\cdot x/L} \text{ and } \omega_k = 2\sum_{m=1}^{d} \sin\left(\frac{\pi k_m}{L}\right).$$

We now consider the local observable $O_n = a_0^\dagger a_0$, and an initial state $|\psi_n(0)\rangle = a_0^\dagger |\text{vac}\rangle$, and provide a lower bound on the error

$$\mathcal{E}_n = \left|\langle\psi_n(0)| e^{iH_nt} O_n e^{-iH_nt} |\psi_n(0)\rangle - \lim_{n\to\infty} \langle\psi_n(0)| e^{iH_nt} O_n e^{-iH_nt} |\psi_n(0)\rangle\right| \quad (35)$$

*Proposition* 1. There exists a nearest-neighbour Hamiltonian $H_n$ on $n = (2L)^d$ fermions (or spins) arranged on a $d-$dimensional lattice, a single-site observable $O_n$ and an initial state $|\psi_n(0)\rangle$ and a time $t$ independent of $n$ such that $\lim_{n\to\infty} \text{Tr}(O_n e^{-iH_nt} \rho_n e^{iH_nt})$ exists and

$$\left|\langle\psi_n(0)| e^{iH_nt} O_n e^{-iH_nt} |\psi_n(0)\rangle - \lim_{n\to\infty} \langle\psi_n(0)| e^{iH_nt} O_n e^{-iH_nt} |\psi_n(0)\rangle\right| \leq \varepsilon \implies n \geq \Omega\left(\frac{\log^d(\Theta(\varepsilon^{-1}))}{\log^d \log(\Theta(\varepsilon^{-1}))}\right).$$

*Proof*: We can obtain an expression for $\langle\psi_n(0)| e^{iH_nt} O_n e^{-iH_nt} |\psi_n(0)\rangle = |\langle\psi_n(0)| e^{-iH_nt} |\psi_n(0)\rangle|^2$ by noting that $|\psi_n(0)\rangle = \frac{1}{(2L)^{d/2}} \sum_{k\in\mathfrak{L}_n^d} \tilde{a}_k^\dagger |\text{vac}\rangle$, and thus,

$$\langle\psi_n(0)| e^{-iH_nt} |\psi_n(0)\rangle = \frac{1}{(2L)^d} \sum_{k\in\mathfrak{L}_n^d} e^{-i\omega_k t} = \frac{1}{(2L)^d} \bigg(\sum_{k=-L}^{L-1} e^{-2it\sin(\pi k/L)}\bigg)^d = \frac{1}{(2L)^d} \bigg(\sum_{k=-L}^{L-1} \cos\left(2t\sin(\pi k/L)\right)\bigg)^d$$

It also follows, from this expression, that

$$\lim_{n\to\infty} \langle\psi_n(0)| e^{-iH_n t} |\psi_n(0)\rangle = \frac{1}{(2\pi)^d}\bigg(\int_{-\pi}^{\pi} \cos(2t\sin\theta)d\theta\bigg)^d$$

Consider now the error $\mathcal{E}_n$ defined in Eq. 35 — from the analytical expression for $\langle\psi_0| e^{-iH_n t} |\psi_0\rangle$ given above, we obtain that

$$\mathcal{E}_n = \frac{1}{(2\pi)^{2d}}\left|\bigg(\int_{-\pi}^{\pi}\cos(2t\sin\theta)d\theta\bigg)^{2d} - \bigg(\frac{\pi}{n}\sum_{k=-n}^{n-1}\cos\big(2t\sin(\pi k/n)\big)\bigg)^{2d}\right|.$$

Note that for $x, y \geq a > 0$ and $k \in \mathbb{N}$, $|x^k - y^k| = |x-y|(x^{k-1} + x^{k-2}y + x^{k-3}y^2 + \dots y^{k-1}) \geq ka^{k-1}|x-y|$. From this fact and from lemma 20, it follows that

$$\mathcal{E}_n \geq \frac{(2d-1)2^d}{(2\pi)^{2d}}\left|\int_{-\pi}^{\pi}\cos(2t\sin\theta)d\theta - \frac{\pi}{n}\sum_{k=-n}^{n-1}\cos(2t\sin(\pi k/n))\right|.$$

Finally, using lemma 21, we obtain that

$$\mathcal{E}_n \geq \frac{(2d-1)2^d}{(2\pi)^{2d-1}}\frac{(\alpha/2)^{2L}}{(2L)!} \geq \frac{(2d-1)2^d}{(2\pi)^{d-1}(2L+1)^{2L+1}}\bigg(\frac{e\alpha}{2}\bigg)^{2L}.$$

Now, if $\mathcal{E}_n \leq \varepsilon$ for small $\varepsilon$, then

$$\bigg(\frac{2L+1}{e\alpha/2}\bigg)^{\frac{2L+1}{e\alpha/2}} \geq \bigg(\frac{2^{d+1}(2d-1)}{e\alpha(2\pi)^{d-1}\varepsilon}\bigg)^{2/e\alpha} \implies (2L+1) \geq \frac{e\alpha}{2}e^{W_0\left(\frac{2}{e\alpha}\log\left(\frac{2^{d+1}(2d-1)}{e\alpha(2\pi)^{d-1}\varepsilon}\right)\right)},$$

where $W_0(\cdot)$ is the Lambert W function. Noting that as $x \to \infty$, $W_0(x) \to \log(x/\log(x))$, we obtain that for $\varepsilon \to 0$ and for a constant $\alpha$, $L \geq \Omega(\log(\Theta(\varepsilon^{-1}))/\log\log(\Theta(\varepsilon^{-1})))$. Since $n = (2L)^d$ we obtain the proposition. □

## B. Ground state

The AKLT Hamiltonian is an example of a Hamiltonian where the convergence of a local observable to the thermodynamic limit is logarithmic and tight [9]. We can also easily construct a higher dimensional model using the AKLT model that satisfies a logarithmic convergence to the thermodynamic limit. Consider a $d$-dimensional lattice $\mathfrak{L}^d = \{0, 1, 2 \dots L-1\}^d$ with $n = L^d$ sites. At each site, we have $d$ spin 1 systems (i.e. the local Hilbert space at each site is $(\mathbb{C}^3)^{\otimes d}$). We consider the following translationally invariant Hamiltonian, with periodic boundary conditions, on this lattice of qudits —

$$H = \sum_{m=1}^{d} H_m \text{ where } H_m = \sum_{x\in\mathfrak{L}^d}\bigg(\vec{S}_x^m\cdot\vec{S}_{x+e_m}^m + \frac{1}{3}\big(\vec{S}_x^m\cdot\vec{S}_{x+e_m}^m\big)^2\bigg), \tag{36}$$

where $\vec{S}_x^m$ is the vector of spin 1 operators at site $x$ and on the $m^{\text{th}}$ spin 1 system at this site, $e_m$ is the unit translation vector on the lattice along the $m^{\text{th}}$ direction. Note that $H_m$ is the sum of 1D AKLT models on the $m^{\text{th}}$ spin in every row of qudits along the $m^{\text{th}}$ direction.

*Lemma* 22. Consider $|G_{L,\text{AKLT}}\rangle = \sum_{i\in\{-1,0,1\}^L}\text{Tr}[A^{i_0}A^{i_1}\dots A^{i_{L-1}}]\,|i_0, i_1 \dots i_{L-1}\rangle$ with $A^{\pm 1} = \pm\sqrt{2/3}\sigma_\pm$, $A^0 = -\sqrt{1/3}\ \sigma_z$, to be the ground state of the 1D AKLT model with periodic boundary conditions on $L$ spins and the single site observable $O_L = |0\rangle\langle 0| \otimes I^{\otimes(L-1)}$ then

$$\langle G_{L,\text{AKLT}}| O_L |G_{L,\text{AKLT}}\rangle = \frac{1}{3}\bigg(1 - \frac{(-1)^{L-1}}{3^{L-1}}\bigg).$$

*Proof*: We can explicitly compute $\langle G_{L,\text{AKLT}}| O_L |G_{L,\text{AKLT}}\rangle$ to obtain

$$\langle G_{L,\text{AKLT}}| O_L |G_{L,\text{AKLT}}\rangle = \frac{1}{3}\sum_{i,j\in\{0,1\}}(-1)^{i+j}\langle i|\,\mathrm{T}^{L-1}(|i\rangle\langle j|)\,|j\rangle\,,$$

where T is the transfer tensor corresponding to the MPS $|G_{L,\text{AKLT}}\rangle$, which is a single qubit channel given by

$$\mathrm{T}(X) = \frac{2}{3}\sigma_+ X\sigma_- + \frac{2}{3}\sigma_- X\sigma_+ + \frac{1}{3}\sigma_z X\sigma_z.$$

We can note that $\mathrm{T}^k(I) = I$, $\mathrm{T}^k(\sigma_z) = (-1/3)^k\sigma_z$, $\mathrm{T}^k(|0\rangle\langle 1|) = (-1/3)^k |0\rangle\langle 1|$, $\mathrm{T}^k(|1\rangle\langle 0|) = (-1/3)^k |1\rangle\langle 0|$. We then obtain that

$$\begin{aligned}
\langle 0|\,\mathrm{T}^k(|0\rangle\langle 1|)\,|1\rangle &= \langle 1|\,\mathrm{T}^k(|1\rangle\langle 0|)\,|0\rangle = \frac{(-1)^k}{3^k},\\
\langle 0|\,\mathrm{T}^k(|0\rangle\langle 0|)\,|0\rangle &= \frac{1}{2}\langle 0|\,\mathrm{T}^k(I)\,|0\rangle + \frac{1}{2}\langle 0|\,\mathrm{T}^k(\sigma_z)\,|0\rangle = \frac{1}{2} + \frac{1}{2}\frac{(-1)^k}{3^k},\\
\langle 1|\,\mathrm{T}^k(|1\rangle\langle 1|)\,|1\rangle &= \frac{1}{2}\langle 1|\,\mathrm{T}^k(I)\,|1\rangle - \frac{1}{2}\langle 1|\,\mathrm{T}^k(\sigma_z)\,|1\rangle = \frac{1}{2} - \frac{1}{2}\frac{(-1)^k}{3^k}.
\end{aligned}$$

Thus,

$$\langle G_{L,\text{AKLT}}|\,O_L\,|G_{L,\text{AKLT}}\rangle = \frac{1}{3}\left(1 - \frac{(-1)^{L-1}}{3^{L-1}}\right),$$

which establishes the lemma. □

*Proposition* 2. There exists a nearest neighbour gapped Hamiltonian $H_n$ on $n$ qudits arranged on a d-dimensional lattice with a unique ground state $|G_n\rangle$, a single site observable $O_n$ such that

$$\left|\langle G_n|\,O_n\,|G_n\rangle - \lim_{n\to\infty}\langle G_n|\,O_n\,|G_n\rangle\right| \leq \varepsilon \implies n \geq \Omega\big(\log^d(\Theta(\varepsilon^{-1}))\big).$$

*Proof*: The Hamiltonian is provided in Eq. 36. Since the AKLT Hamiltonian is known to be gapped, this Hamiltonian is gapped as well. We choose the observable $O_n = (|0\rangle\langle 0|)^{\otimes d} \otimes I^{\otimes(n-1)}$ i.e. the projector $|0\rangle\langle 0|$ on all the spin 1 systems at the first site of the lattice, and identity on the remaining sites. Using lemma 22, we obtain that

$$\langle G_n|\,O_n\,|G_n\rangle = \frac{1}{3}\left(1 - \frac{1}{3^{n^{1/d}-1}}\right)^d,$$

where, for simplicity, we assume that $n^{1/d}$ is odd. We then obtain that

$$\left|\langle G_n|\,O_n\,|G_n\rangle - \lim_{n\to\infty}\langle G_n|\,O_n\,|G_n\rangle\right| = \frac{1}{3}\left(1 - \left(1 - \frac{1}{3^{n^{1/d}-1}}\right)^d\right) \leq \varepsilon \implies n \geq \Omega\big(\log^d(\Theta(\varepsilon^{-1}))\big),$$

which establishes the proposition. □

### C. Fixed points

Glauber dynamics for classical Ising models are examples of Lindblad evolutions which satisfy rapid mixing, and the convergence of local observables to the thermodynamic limit is logarithmic and tight. To construct an explicit example, we begin with the following result, which follows directly from the transfer matrix method.

*Lemma* 23. Consider $L$ spins on 1D lattice in the state $\rho_L = e^{-\beta H_L}/\mathrm{Tr}[e^{-\beta H}]$ where $H_L = -\sum_{i=1}^{L-1} Z_i Z_{i+1}$. Let $\langle Z_1 Z_2\rangle_L := \mathrm{Tr}(Z_1 Z_2 \rho_L)$, then

$$\frac{\tanh^{L-2}\beta}{\sqrt{2}\sinh\beta\cosh\beta} \leq \left|\langle Z_1 Z_2\rangle_L - \lim_{L\to\infty}\langle Z_1 Z_2\rangle_L\right| \leq \frac{\sqrt{2}\tanh^{L-2}\beta}{\sinh\beta\cosh\beta}.$$

*Proof.* The proof of this proposition follows by explicit computation using the transfer matrix method [10]. We note that

$$\mathrm{Tr}(e^{-\beta H}) = \sum_{\sigma\in\{-1,1\}^L} e^{\beta\sigma_1\sigma_2}e^{\beta\sigma_2\sigma_3}\ldots e^{\beta\sigma_{L-1}\sigma_L} = \mathrm{Tr}\big(\mathrm{T}^{L-1}\big),$$

and

$$\mathrm{Tr}(Z_1 Z_2 e^{-\beta H}) = \sum_{\sigma \in \{-1,1\}^L} \sigma_1 \sigma_2 e^{\beta \sigma_1 \sigma_2} e^{\beta \sigma_2 \sigma_3} \dots e^{\beta \sigma_{L-1} \sigma_L} \mathrm{Tr} = \big(\mathrm{R}\mathrm{T}^{L-2}\big),$$

where

$$\mathrm{T} = \begin{bmatrix} e^{\beta} & e^{-\beta} \\ e^{-\beta} & e^{\beta} \end{bmatrix} \text{ and } \mathrm{R} = \begin{bmatrix} e^{\beta} & -e^{-\beta} \\ -e^{-\beta} & e^{\beta} \end{bmatrix}.$$

Therefore, we obtain an explicit expression for $\langle Z_1 Z_2 \rangle_L$:

$$\langle Z_1 Z_2 \rangle_L = \frac{\mathrm{Tr}\big(\mathrm{R}\mathrm{T}^{L-2}\big)}{\mathrm{Tr}\big(\mathrm{T}^{L-1}\big)} = \frac{\sqrt{2}\big(\sinh\beta \cosh^{L-2}\beta + \cosh\beta \sinh^{L-2}\beta\big)}{\cosh^{L-1}\beta + \sinh^{L-1}\beta}.$$

We note that for any $\beta > 0$, $\cosh\beta > \sinh\beta$ and therefore $\lim_{L\to\infty} \langle Z_1 Z_2 \rangle_L = \sqrt{2}\tanh\beta$. Furthermore, from the explicit expression for $\langle Z_1 Z_2 \rangle_L$, we obtain that

$$\Big|\langle Z_1 Z_2 \rangle_L - \lim_{L\to\infty} \langle Z_1 Z_2 \rangle_L\Big| = \frac{\sqrt{2}}{\sinh\beta\cosh\beta}\left|\frac{\sinh^{L-2}\beta}{\sinh^{L-2}\beta + \cosh^{L-2}\beta}\right|.$$

From this explicit expression and observing that $\cosh^{L-2}\beta \leq \cosh^{L-2}\beta + \sinh^{L-2}\beta \leq 2\cosh^{L-2}\beta$, we obtain the lemma statement.

*Proposition* 3. There exists a nearest neighbour Lindbladian $\mathcal{L}_n$ on $n$ qudits arranged on a d-dimensional lattice with a unique fixed point $\sigma_n$ and which satisfies rapid mixing, and a local observable $O_n$ such that

$$\Big|\mathrm{Tr}\big(\sigma_n O_n\big) - \lim_{n\to\infty} \mathrm{Tr}\big(\sigma_n O_n\big)\Big| \leq \varepsilon \implies n \geq \Omega\big(\log^d(\Theta(\varepsilon^{-1}))\big).$$

*Proof.* Let us first consider the 1D setting — a spatially local rapidly mixing Lindbladian $n$ spins, $\mathcal{L}_n^{(1)} = \sum_{\alpha=0}^{n-2} \mathcal{L}^{(1)}_{\alpha,\alpha+1;n}$ where $\mathcal{L}^{(1)}_{\alpha,\alpha+1;n}$ acts on $\alpha^{\text{th}}, (\alpha+1)^{\text{th}}$ spins, and a local observable that proves the proposition can be constructed by a quantum encoding of classical Glauber dynamics preparing the Gibbs state (at any non-zero inverse temperature $\beta$) of the classical Ising model $H = -\sum_{i=1}^n Z_i Z_{i+1}$. This construction is explicitly outlined in Ref. [8] and shown to be rapidly mixing *as a quantum evolution* as a consequence of log Sobolev inequalities that have been established for the classical Glauber dynamics [11]. Furthermore, from lemma 23, it follows that the local observable $Z_0 Z_1$ satisfies the convergence condition in the proposition statement.

Similar to the example of the gapped ground state, the 1D example can then be used to construct the $d$-dimensional example. We consider spins arranged on the $d-$dimensional lattice $\mathbb{Z}_L^d$ where $L = n^{1/d}$ — at every lattice site, we will have $d$ spins (i.e. the local Hilbert space is $\big(\mathbb{C}^2\big)^{\otimes d}$). We will consider the nearest-neighbour Lindbladian formed by implementing the Lindbladian

$$\mathcal{L}_n^{(d)} = \sum_{m=0}^{d-1} \mathcal{L}^{(d)}_{m;n} \text{ where } \mathcal{L}^{(d)}_{m;n} = \sum_{\alpha \in \{0,2\dots L-2\}^d} \mathcal{L}^{(1)}_{\alpha_m,\alpha_m+1} \otimes \mathrm{id},$$

where $\mathcal{L}^{(1)}_{\alpha_m,\alpha_m+1}$ acts on the $m^{\text{th}}$ spin at site $\alpha$ and the $m^{\text{th}}$ spin at the site displaced by one unit from $\alpha$ along the $m^{\text{th}}$ direction. We point out that since $\mathcal{L}_n^{(1)}$ satisfies rapid mixing, by construction, so does $\mathcal{L}_n^{(d)}$. Consider now the observable $O_n$

$$O_n = \prod_{m=0}^{d-1} Z_{0,m} Z_{e_m,m},$$

where $Z_{\alpha,m}$ is the $Z$ operator acting on the $m^{\text{th}}$ spin at site $\alpha$, amd $e_m$ is the unit vector along the $m^{\text{th}}$ lattice direction. Using lemma 23, it can be seen that

$$\mathrm{Tr}(\sigma_n O_n) = \big(\langle Z_1 Z_2 \rangle_{n^{1/d}}\big)^d,$$

where $\langle Z_1 Z_2\rangle_L$ is defined in lemma 23. Note that $\lim_{n\to\infty} \mathrm{Tr}(\sigma_n O_n) = (\sqrt{2}\tanh\beta)^d$. We also obtain from lemma 23 that for positive $\beta$ which is $\Theta(1)$ ,

$$\langle Z_1 Z_2\rangle_{n^{1/d}} \geq \sqrt{2}\tanh(\beta) - O(e^{-\Theta(n^{1/d})}) \text{ and } \left|\langle Z_1 Z_2\rangle_{n^{1/d}} - \sqrt{2}\tanh\beta\right| \geq \Omega(e^{-\Theta(n^{1/d})}).$$

We now obtain that

$$\left|\mathrm{Tr}(\sigma_n O_n) - \lim_{n\to\infty}\mathrm{Tr}(\sigma_n O_n)\right| = \left|\langle Z_1 Z_2\rangle_{n^{1/d}} - \sqrt{2}\tanh\beta\right| \times \sum_{j=0}^{d-1}\langle Z_1 Z_2\rangle^j_{n^{1/d}}\big(\sqrt{2}\tanh\beta\big)^{d-1-j},$$
$$\geq \Omega(e^{-\Theta(n^{1/d})}) \times \big(\sqrt{2}\tanh(\beta) - O(e^{-\Theta(n^{1/d})})\big) \geq \Omega(e^{-\Theta(n^{1/d})}),$$

from which the proposition follows.


[1] Rajendra Bhatia. *Matrix analysis*, volume 169. Springer Science & Business Media, 2013.

[2] Matthew B Hastings. Lieb-schultz-mattis in higher dimensions. *Physical review b*, 69(10):104431, 2004.

[3] Sergey Bravyi, Matthew B Hastings, and Frank Verstraete. Lieb-robinson bounds and the generation of correlations and topological quantum order. *Physical review letters*, 97(5):050401, 2006.

[4] Sven Bachmann, Spyridon Michalakis, Bruno Nachtergaele, and Robert Sims. Automorphic equivalence within gapped phases of quantum lattice systems. *Communications in Mathematical Physics*, 309(3):835–871, 2012.

[5] David Poulin and Pawel Wocjan. Sampling from the thermal quantum gibbs state and evaluating partition functions with a quantum computer. *Physical review letters*, 103(22):220502, 2009.

[6] Fernando GSL Brandão and Michael J Kastoryano. Finite correlation length implies efficient preparation of quantum thermal states. *Communications in Mathematical Physics*, 365(1):1–16, 2019.

[7] Matthew B Hastings. Quantum belief propagation: An algorithm for thermal quantum systems. *Physical Review B*, 76(20):201102, 2007.

[8] Toby S Cubitt, Angelo Lucia, Spyridon Michalakis, and David Perez-Garcia. Stability of local quantum dissipative systems. *Communications in Mathematical Physics*, 337:1275–1315, 2015.

[9] Ian Affleck, Tom Kennedy, Elliott H Lieb, and Hal Tasaki. Rigorous results on valence-bond ground states in antiferromagnets. *Condensed Matter Physics and Exactly Soluble Models: Selecta of Elliott H. Lieb*, pages 249–252, 2004.

[10] Theodore D Schultz, Daniel C Mattis, and Elliott H Lieb. Two-dimensional ising model as a soluble problem of many fermions. *Reviews of Modern Physics*, 36(3):856, 1964.

[11] Fabio Martinelli. Lectures on glauber dynamics for discrete spin models. *Lectures on probability theory and statistics (Saint-Flour, 1997)*, 1717:93–191, 1999.
